# A static VM placement and hybrid job scheduling model for green data centers

**DOI:** 10.1371/journal.pone.0237238

**Published:** 2020-08-13

**Authors:** Zahra Movahedi Nia, Mohammad Reza Khayyambashi, Ali Miri

**Affiliations:** 1 Faculty of Computer Engineering, University of Isfahan, Isfahan, Iran; 2 Department of Computer Science, Ryerson University, Toronto, Canada; Sunway University, MALAYSIA

## Abstract

Reducing energy consumption has become a critical issue in today data centers. Reducing the number of required physical and Virtual Machines results in energy-efficiency. In this paper, to avoid the disadvantages of VM migration, a static VM placement algorithm is proposed which places VMs on hosts in a Worst-Fit-Decreasing (WFD) fashion. To reduce energy consumption further, the effect of job scheduling policy on the number of VMs needed for maintaining QoS requirements is studied. Each VM is modeled by an M/M/* queue in space-shared, time-shared, and hybrid job scheduling policies, and energy consumption of real-time as well as non-real-time applications is analyzed. Numerical results show that the hybrid policy outperforms space-shared and time-shared policies, in terms of energy consumption as well as Service Level Agreement (SLA) violations. Moreover, our non-migration method outperforms three different algorithms which use VM migration, in terms of reducing both energy consumption and SLA Violations.

## 1. Introduction

Ever-increasingly, companies move to the cloud to lower their budgets and reduce costs by benefiting from pooled hardware and software resources, delivered as IT services. These shared, on demand services are mainly offered in three forms: Infrastructure as a Service (IaaS), Platform as a Service (PaaS), and Software as a Service (SaaS). In the scope of IaaS clouds (e.g., Amazon EC2, Microsoft Azure, and Google Compute Engine (GCE)), server virtualization is a key factor for elasticizing the data center by sharing computing resources between several users and simultaneously, isolating them from each other. Elasticity, the ability to expand and contract resources as workloads change, is an important aspect of IaaS clouds, which profits both cloud providers and users by removing the extra resources. Elasticity not only decreases green-house gasses and global warming by reducing the energy consumption of data centers, but also cuts the costs for cloud users, while providing sufficient resources to maintain QoS requirements. Server virtualization benefits IaaS datacenters, in fundamental different ways [1]:

- Physical servers are usually too big for a single user. Virtual machines can divide them into smaller virtual servers, and assign each one to a different user.
- The Virtual Machine (VM) technology is capable of isolating different users and workloads from each other, while sharing hardware resources between them.
- Virtual servers are easier to manage because they are software-based and expose a uniform interface through standard abstractions.

QoS requirements and obligations such as response time, reliability, availability, and security are summarized in Service Level Agreement (SLA), and a penalty is charged for any violation [2]. In this paper, we focus on response time and avoid SLA violations by verifying that the time it takes for a request to complete does not surpass its deadline. It is justified in [3] that focusing on CPU usage is sufficient in managing the resource allocations to CPU-intensive applications. So, we concentrate on CPU usage, and propose a method to reduce the amount of resources allocated to services while maintaining their SLA requirements. Basically, servers consume about 45% of the whole energy of a data center while their energy consumption is not proportional to their load [4]. As a server turns on, it consumes 69–97% of its total energy, therefore, when the server is idle or under-loaded, much of its energy is literally wasted [5]. Thus, one way to reduce the energy consumed in a data center is to turn off as many servers as possible. But as it takes a considerable amount of time to turn on a switched off host, so, it is wise to switch an idle host into a sleeping mode instead of turning it off.

Some papers consider using VM migration to dynamically place as many VMs as possible on a server and reduce the number of active servers [6, 7]. VM migration could be very time consuming and detrimental to real-time applications, therefore, live migration is used to reduce the downtime. Live VM migration is the practice of moving a running VM from one physical machine to another, without disconnecting the applications. Although, live migration can decrease energy consumption, it introduces some additional energy usage to the data center. It has been shown in [8] that static resource allocation is more energy-efficient than dynamic allocation. VM migration (even in its live form) brings many disadvantages. First of all, it hurts real-time applications, as it increases latency. It also causes difficulty for the addressing and routing system, e.g., when the VM migrates to a new subnet, its IP address has to be changed, and subsequently, all the forwarding tables in the switches and routers need to be modified. Moreover, it unexpectedly intensifies the traffic and might cause congestion. As such, some of the previously proposed methods completely avoid VM migration, but usually require the workload to be predictable [9–11].

In this paper, we propose a method to place VMs on less number of servers statically in a Worst-Fit-Decreasing (WFD) fashion. WFD is a bin packing algorithm that according to our results performs better than other bin packing algorithms, namely, First-Fit-Decreasing (FFD) and Best-Fit-Decreasing (BFD) when placing VMs on servers. To further make our method more energy-efficient, we purpose a hybrid job scheduling algorithm using queuing theory that reduces the number of VMs by placing as many jobs as possible on a VM. By using less VMs, we utilize less servers and eventually reduce energy consumption.

Generally, Virtual Machines (VM) use two main scheduling policies, namely, space- and time-shared to assign resources to the jobs appointed to them. Additionally, hybrid algorithms, combine the two aforementioned policies. While in space-shared, which is non-preemptive, the processing space is shared among different jobs, in time-shared, which is pre-emptive, time is divided between them [12, 13]. In hybrid mode, both time and processing space are divided between jobs. A good comparison between different algorithms of each class of policies is given in [13]. Basically, First-Come-First-Serve (FCFS) and Shortest-Job-First (SJF) are the two main algorithms used for space-shared policy. Another approach to space-sharing is randomization which unfavorably, eliminates predictability. Priority scheduling, another space-shared method, processes jobs according to certain predefined priority levels. Moreover, backfilling, in which shorter jobs can be processed in the unused spaces left between longer ones, is another technique used in space-shared policy [14]. In a time-shared policy, the scheduler shifts between jobs in different time slices. One of the simplest algorithms for this discipline is Round Robin (RR), which treats all jobs equally. In contrast, Shortest Remaining Time (SRT) and Highest Response Ratio Next (HRRN), always choose the job that has the shortest processing time left and the job with highest response ratio ((*W* + *T*_*s*_)/*T*_*s*_, where *W* is the job’s waiting time and *T*_*s*_ is the expected service time), for the next slice, respectively. Feedback scheduling is another pre-emptive scheduling algorithm which concentrates on the time spent in execution so far, rather than the remaining time to execute. The hybrid mode includes algorithms such as co-operative scheduling and scheduling with Message Passing Interface (MPI). Detailed discussion of job scheduling disciplines is beyond the scope of this paper.

Inspired with static VM allocation superiority, in this paper, we propose a non-migration method which does not necessarily need to predict the workload, when placing VMs on hosts in a WFD fashion. As an extension to our research and to reduce the energy consumption further, a novel queue-based algorithm is proposed to schedule jobs on fewer number of VMs by finding the right number of VMs required for each cloud user to maintain SLA conditions. By removing the extra VMs, we aim to utilize fewer number of servers and reduce energy consumption. Since a VM with space-share policy shares the processor space between jobs non-preemptively, it executes the jobs on different cores of the processor. Therefore, it is modeled by an M/M/m queue. Normally, all the cores of a processor are the same and have equal capacity. Furthermore, we make sure jobs are assigned to the VMs which have enough capacity for executing them. In such a situation, FCFS with backfilling, which performs very well [15] and even near-optima [16], converges to FCFS, as there is no wholes left between longer jobs [15]. In addition, FCFS is the fairest space-shared policy [17, 18]. Other disciplines such as SJF, randomization, and priority scheduling are prone to starvation. To this end, a VM with space-shared policy is modeled by an M/M/m/FCFS queue.

In time-shared policy, since Processor Sharing (PS) discipline is a good approximation for RR algorithm, a VM is modeled by an M/M/1/PS queue. In hybrid mode, each core devotes a time slot to a different job in an RR manner. Therefore, it is modeled by an M/M/m/PS queue. In each scenario, the maximum request arrival rate *λ*_*max*_ of each VM is derived. By modeling SLA in terms of response times or latencies, both real-time and non-real-time applications are taken into consideration. Our findings show that there is a trade-off between server utilization and SLA violation: The busier a server gets, the more time is required for each job to be completed. Analyzing VM request queues, we balance those two metrics to design an energy-aware non-migration algorithm for placing VMs on servers in a WFD bin-packing fashion, as WFD is found in our work to have better performance in placing VMs on physical hosts compared with the other two algorithms, namely BFD and FFD bin-packing.

To simulate our algorithm CloudSim [12] is interfaced with Matlab. We found that the hybrid mode, remarkably, outperforms space-shared and time-shared policies and assembles their advantages. We conclude that by applying hybrid policies in conjunction with non-migration VM management, SLA conditions are better maintained, and less energy is consumed. The simulation results show that our non-migration method outperforms three different algorithms which use VM migration in terms of reducing both energy consumption and SLA violation.

The remainder of the paper is organized as follows. Related work is reviewed in Section 2. Section 3 and 4 describe our proposed model. Section 5 presents the experimental results. A conclusion is made in section 6.

## 2. Related work

To reduce the power consumption, the proposed methods in [6] and [7] decrease the number of active servers. The authors of [19] propose a method called PRESS in which an elastic resource allocation scheme is developed by predicting tenants’ behavior. The prediction is done using Fast Fourier Transform (FFT) for workloads which do have a repeating pattern and discrete time Markov chain for workloads that do not. In [20], a cloud server farm is modeled by an M/M/R queue. Then, a cost function consisting of system operating cost, working mode cost, and system congestion cost is minimized using two heuristic algorithms called OCP, and OCPF. Although [19] and [20] present a model to reduce energy consumption, they do not propose any method for finding the optimal number of VMs or placing them on servers. In [21], the M/M/1 Queuing Theory Predicting Method (MMQMPM) is improved for predicting the workload. It argues that the delay of MMQMPM, during the rapidly increasing period is unacceptable. To overcome this problem, it uses the Linear Trend Predicting Method (LTPM), which responds quickly to continuously increasing sequences. In [22] and [23] resources are modeled by an M/M/c and M/M/1 queues. They both formulate an optimization problem for resource provisioning and reducing power consumption. Authors in [24] model an incoming data stream by a queue with arrival rate *λ*. In order to satisfy QoS requirements, they define a limit on maximum waiting times, corresponding to deadlines. By Little’s law, they transfer this limit to an upper bound on the queue’s length. Using this upper bound, they adjust the number of VMs to reduce energy consumption.

The reported works in [25–27] try to reduce the number of migrations, but they didn’t completely avoid VM migration, as it requires the workload to be predictable. However, our proposed algorithm does not necessarily need to foresee the workload. The authors of [9] bring two scenarios under consideration: SSAP which represents a constant workload over time, and SSAPv which provides cyclic workloads over time. For each of these scenarios an optimization problem is formulated and then solved by Branch & Bound and heuristic algorithms, and then compared with each other. Similarly, in [10] SSAP and SSAPv have been formulated as an optimization problem and solved by heuristic algorithms. In [11], considering most workloads are seasonally cyclic, the constraint matrix of each VM is derived by analyzing resource demands over time, and then reduced using Singular Value Decomposition (SVD). Using the information gained, an integer programming problem is formulated to optimally place VMs on servers. In this paper we build a paradigm to implement energy-aware non-migration VM placement, which do not necessarily need the workload to be predictable.

The authors of [9] and [28] use FFD algorithms to find the minimum number of servers required for placing a certain amount of VMs on them. Whereas [29] uses BFD to minimize the number of required servers for VM accommodation. However, [30] and [31] found that WFD has performance in placing VMs on physical hosts. In [30], the placement of jobs on VMs, and VMs on hosts has been organized, using bin packing algorithms. Accordingly, four different strategies have been examined: best fit-best fit, worst fit-worst fit, worst fit-best fit, best fit-worst fit. It is concluded that placing the jobs on VMs by best fit, and VMs on hosts by worst fit algorithms (best fit-worst fit), present the best results for reducing energy consumption. In [31], simulated annealing has been combined with worst-fit decreasing algorithm to solve the bin-packing problem. We use WFD to implement our energy-aware non-migration algorithm and compare it with WFD, BFD, and FFD which use VM migration for optimizing VM placement.

None of the works explained above consider the VM’s job scheduling policy, when rightsizing the allocated resources. Moreover, they do not bring real-time applications under consideration. EARH [32] proposes a scheduling paradigm to manage energy consumption for real-time applications. It utilizes a rolling horizon architecture to schedule jobs with respect to their deadlines. SEATS [33] provides equations to obtain the CPU utilization point of a host where energy consumption is minimum. According to that it places the VMs on the hosts in a way that real-time tasks are executed before their deadlines. In [34] Markov queuing theory is used to schedule tasks on VMs according to their SLA conditions. Then, an optimization problem is formulated to reduce energy consumption while placing VMs on hosts. Four different heuristic algorithms are used to solve the optimization problem. Although papers [32–34] use VM migration to decrease energy consumption, their focus aims at avoiding the side effects, especially on real-time applications. In two rather similar but separate works, ant colony optimization is used for virtual machine placement in data centers. Both works have reported that their method has outperformed FFD in terms of power consumption [35, 36]. To optimize energy consumption in dynamic VM placement, a partitioned optimization method is proposed in [37] by combining the partheno-genetic algorithm with a multiplayer random evolutionary game theory to find the best migration possibility. As most works propose a method for non-real-time applications, real-time applications are considered rarely.

In this paper, we propose an energy-aware non-migration VM placement algorithm which benefits real-time applications the most. We show that taking the job scheduling policy into consideration and adopting hybrid policy instead of space-shared or time-shared can remarkably reduce the energy consumption while maintaining SLA conditions. For this purpose, a resource-rightsizing algorithm for a space-shared policy with FCFS and a time-shared policy with RR discipline, and hybrid policy: the combination of space-shared and time-shared policies, are tested. We take both real-time and non-real-time applications into consideration. The results show that the hybrid policy excels space-shared and time-shared policies lower energy consumption and fewer SLA violations. In addition, the hybrid policy outperforms WFD, BFD, and FFD with VM migration, in terms of reducing both energy consumption and SLA violation. The next two sections explain our proposed model. The terminology used is summarized in Table 1.

**Table 1:** Terminology.

Symbol	Indication
***N*_*max*_**	Maximum Number of Jobs Allowed in a VM
***λ*_*max*_**	Maximum Acceptable Mean Arrival Rate
***T*_*s*_**	Mean Service time
***μ***	Mean Service Rate
***W***	Waiting Time
***ρ***	Utilization Factor
t	A Random Variable on the Sojourn Time
***μ*_*t*_**	Expected Value of the Sojourn Time
***σ*_*t*_**	Standard Deviation of the Sojourn Time
$\hat{T}$	Maximum Acceptable Sojourn Time
***Pr***(.)	Probability Function
$\alpha ,\;\hat{\alpha }$	Smoothing Factors
m	Number of Pes
n	Number of Requests Waiting for Service
***π*_*0*_**	Probability of No Request Waiting in the Queue
***π*_*w*_**	Probability of Requests Waiting for Service in the Queue
$M_{0}^{\left(1\right)}$	First Moment of No Request Waiting in the Queue
$M_{0}^{\left(2\right)}$	Second Moment of No Request Waiting in the Queue
b	Probability Bound

### 3. The proposed model

According to what has been stated in the SLA, some minimum number of VMs are initially assigned to each cloud user. It has been shown in [30] and [31] that among BFD, FFD, and WFD, WFD performs better. Thus, we use WFD in our non-migration VM management strategy to place these VMs on data center servers. In WFD, first, the hosts and the VMs are sorted according to the amount of processor capacity they respectively have and require in decreasing order. Then the largest VM is placed on the largest host. The next VMs are in turn placed on the largest available space on the utilized hosts. If a VM could not fit on the largest available space, a new server is utilized.

**Fig 1:**
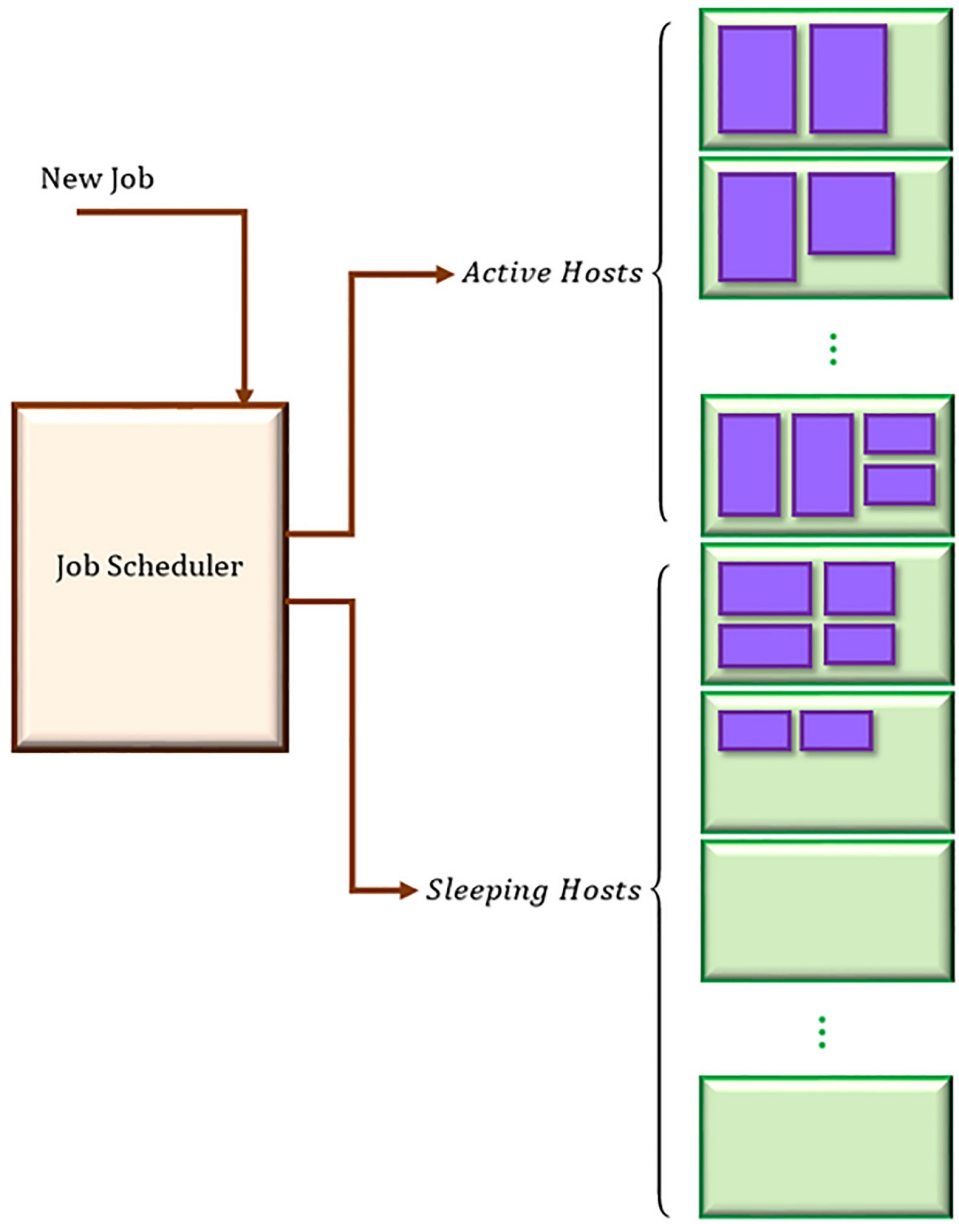
The proposed model.

We aim to execute the jobs on minimum number of VMs possible, and therefore, reduce the number of active hosts. Since it takes considerable amount of time to turn on a server, a host whose all its VMs are idle will not be switched off, instead, it will be asleep. This is extremely useful for real-time applications. As depicted in Fig 1, when a new job arrives, the job scheduler faces two different scenarios: (1) there is an active VM with enough capacity to execute it, (2) all of the active VMs are fully occupied and cannot undertake the job, and therefore, a new VM should be activated or created.

In our method, when a job is executed and completed, the VM updates the maximum request arrival rate, *λ*_*max*_ (We will see in section 4 that to calculate *λ*_*max*_, the mean execution time, *T*_*s*_, is required. Thus, when a job is completed, *λ*_*max*_ needs to be updated.).

The job scheduler unit keeps a record for each VM, stating whether the VM can or cannot accept more jobs. The job scheduler uses this record to decide whether or not it should give a newly arrived job to a VM. Each time a new job is assigned to a VM, or completed and removed, the VM sends a report to the job scheduler unit and updates its record. Algorithm 1 shows a VM sending a report to the job scheduler unit upon a new job arriving, or a job departing. In this algorithm, the VM uses Little’s law to convert the maximum request arrival rate *λ*_*max*_ to the maximum number of jobs in the VM, *N*_*max*_ (*N*_*max*_ = 1 + *λ*_max_.*waiting_time* where *λ*_*max*_.*waiting_time* number of jobs are waiting for service and 1 job is being served). If the number of jobs that the VM is serving is smaller than *N*_*max*_, the VM reports to the job scheduler that it “can accept more jobs” otherwise, it reports that it “cannot accept more jobs”.

**Algorithm 1**. **VM Report Upon a New Job Arriving or a Job Departing**

Input: *λ*_max_, the maximum request arrival rate the VM is allowed to have;

*W*, maximum waiting time.

job_number: the number of jobs being served by this VM.

Output: Report stating whether it can accept more jobs or not.

 1: *N*_*max*_ = *λ*_*max*_.*W* + 1

 2: if *job*_*number*_ < *N*_*max*_ then

 3: report = can_accept_more_jobs

 4: else

 5: report = cannot_accept_more_jobs

 6: send (report)

When the service is entering busy hours, or the job request rate is increasing, the job scheduler unit might find that none of the active VMs are able to undertake the new job, therefore, a new VM should be activated or created. We designed the job scheduler unit to make a fair decision in the four following steps:

1. **If there are idle sleeping VMs on active hosts, activate the largest one and assign the new job to it**. The host is already active and much of its energy is being consumed. By activating the largest VM, we make better use of the host and provide more processing capacity to the increasing number of arriving jobs.
2. **If there isn’t any idle VM on the active hosts, but there is enough empty space on them, select the largest space and create the largest VM which fits in that space**. Aside from making better use of active hosts and presenting biggest processing capacity possible to the increasing number of arriving jobs, this decision is the closest to WFD policy.
3. **If no VM can be activated or created on the active hosts, but there are idle VMs on sleeping hosts, select the smallest sleeping host and activate the largest VM on it**. Since we are activating only one VM of a host, a great portion of the hosts’ CPU capacity will be unused. With a larger host, which consumes more energy, more CPU capacity is unused and wasted. Additionally, the largest VM consumes a greater portion of hosts’ processing capacity and provides greater processing capacity to the increasing number of arriving jobs.
4. **If no VM can be activated or created on the active hosts, and there is no idle VM on the sleeping ones, select the smallest host and create the largest VM on it**. Other than the reason explained in the previous part, this decision is the closest to WFD policy.

The job scheduler unit checks the VM reports in the order of VM creation. In other words, the VMs which were created earlier are checked sooner. This way, when the busy hours are over, or the job request rate is decreasing, new jobs are assigned to older VMs and the younger ones would gradually become idle and asleep in turn. A host can go to sleep as soon as all of its VMs are idle. Once a VM is created, we do not delete it anymore, because it is very likely that it would be needed again soon when the job request rate increases. With this paradigm, we place the VMs on servers in a WFD fashion, and reduce the number of servers used and the amount of energy consumed without migrating any VM. We elasticize the data center by adding and removing VMs from the service. In other words, we divided the VMs into two parts: (1) the minimum number of VMs which are almost always running, because the load seldom gets lower than the sum of their capacities, and (2) additional VMs, which are added to the service as the load increases, and removed again when the load decreases. The minimum number of VMs are created initially and located on the servers according to WFD algorithm. The additional VMs are located on the hosts later on in our aforementioned four steps with a policy very close to WFD. Algorithm 2 provides the code of the job scheduler unit. In the next section, we explain how VMs calculate their maximum request arrival rate *λ*_*max*_.

**Algorithm 2**. **The job scheduler unit**

Input: VM, a list of all VMs in the order of their age, stating whether they can accept more jobs or not,

   active_hosts, a list of active hosts,

   sleeping_hosts, a list of sleeping hosts.

Output: A suitable VM for the new job.

 1: selected_vm = null

 2: **for each** vm **in** VM **do**

 3:  **if** report(vm) = = can_accept_more_jobs **then**

 4:   selected_vm = vm

 5:   break;

 6:  **end if**

 7: **end for**

 8: **if** selected_vm = = null **then**

 9:    largest_idle_vm = null

 10:  **for each** host **in** active_hosts **do**

 11:   **for each** idle_vm **in** host **do**

 12:    **if** largest_idle_vm = = null **or** largest_idle_vm < idle_vm **then**

 13:     largest_idle_vm = idle_vm

 14:    **end if**

 15:   **end for**

 16:  **end for**

 17:  selected_vm = largest_idle_vm

 18: **end if**

 19: **if** selected_vm = = null **then**

 20:  largest_idle_vm = null

 21:  **for each** host **in** sleeping_hosts **do**

 22:   **for each** idle_vm **in** host **do**

 23:    **if** largest_idle_vm = = null **or** largest_idle_vm < idle_vm **then**

 24:     largest_idle_vm = idle_vm

 25:    **end if**

 26:   **end for**

 27:  **end for**

 28:  selected_vm = largest_idle_vm

 29: **end if**

 30: **if** selected_vm = = null **then**

 31:  smallest_host = null

 32:  **for each** host **in** sleeping_hosts **do**

 33:   **if** smallest_host = = null **or** smallest_host > host **then**

 34:    smallest_host = host

 35:   **end if**

 36:  **end for**

 37:  selected_vm = create_largest_vm (smallest_host)

 38: **end if**

 39: **return** selected_vm

## 4. The maximum request arrival rate, *λ*_*max*_

We define a start time, an execution time, and a deadline for each job. Therefore, the waiting time is calculated by:

W=deadline-starttime-executiontime (1)

In real-time applications, *W* is as small as 100 (ms), but in non-real-time procedures, it can be greater [38]. Since requests are submitted stochastically, we can assume that arrival times have an exponential distribution with mean arrival rate *λ*. In fact, it is very common to assume the arrival time and service time of the jobs have an exponential distribution [20–24]. As the mean request arrival rate, *λ*, increases, the VM gets busier and it takes more time for each job to complete. Our goal is to discover the maximum value of *λ*, which keeps the time jobs spend in the VM lower than *W* and no SLA violation occurs. Taking *t* as a random variable denoting the time a job spends in the VM (sojourn time), we can find a lower bound on the probability $Pr(t\leq \hat{T})$ using Cantelli’s inequality which is a generalization of Chebyshev’s inequality [39]

$$Pr\;(t-\mu_{t}\geq h)\left\{ \begin{array}{c} \leq \frac{\sigma_{t}^{2}}{\sigma_{t}^{2}+h^{2}}\;,h>0\\ \geq 1-\frac{\sigma_{t}^{2}}{\sigma_{t}^{2}+h^{2}}\;,h<0 \end{array}\right.$$ (2)

Where *μ*_*t*_ and $\sigma_{t}^{2}$ are the mean and variance of *t*, respectively, *h* can be any real number, and $\hat{T}$ is the maximum sojourn time possible

$$\hat{T}\;=\;W+execution\;time$$ (3)

Assuming $\hat{T}=h+\mu_{t}\Rightarrow h=\hat{T}-\mu_{t}$, and since $h=\hat{T}-\mu_{t}>0$, we can have

$$Pr(t-\mu_{t}\geq h)=Pr\;(t\geq h+\mu_{t})\leq \frac{\sigma_{t}^{2}}{\sigma_{t}^{2}+h^{2}}$$ (4)

Thus,

$$Pr(t\geq \hat{T})\leq \frac{\sigma_{t}^{2}}{\sigma_{t}^{2}+{\left(\hat{T}-\mu_{t}\right)}^{2}}⟹Pr(t\leq \hat{T})\geq \frac{{\left(\hat{T}-\mu_{t}\right)}^{2}}{\sigma_{t}^{2}+{\left(\hat{T}-\mu_{t}\right)}^{2}}$$ (5)

In the following sections, our models for space-shared, time-shared, and hybrid policies are described.

### 4.1. Space-shared policy

In space-shared policy the VM shares the CPU cores between jobs, non-preemptively [12]. A VM with space-share policy is modeled by an M/M/m/FCFS queue. We denote the arrival rate by *λ* and the mean service time by *T*_*s*_ = 1/(*mμ*). Thus, the utilization factor is *ρ* = *λ*/(*mμ*) = *λT*_*s*_. According to Eq (5), in order to find a lower limit on $Pr(t\leq \hat{T})$, we need the mean and variance of *t*, which can be obtained for FCFS discipline by Eqs (6) and (7) [40].

$$\mu_{t}\;=\;\frac{1}{\mu }+\frac{\pi_{w}}{m\mu (1-\rho )}$$ (6)

$$\sigma_{t}^{2}\;=\;\frac{1}{\mu^{2}}+\frac{2\pi_{w}-\pi_{w}^{2}}{{\left(m\mu (1-\rho )\right)}^{2}}$$ (7)

Where *π*_*w*_ is the probability of the number of requests waiting in the queue (*n*), being greater or equal to m. In other words, the probability that a request must wait for service, when it arrives.

$$\pi_{w}\;=\;Pr(n\geq m)\;=\;\frac{{\left(m\rho \right)}^{m}\pi_{0}}{m!(1-\rho )}$$ (8)

$$\pi_{0}=Pr(n=0)={\left(\sum_{j=0}^{m-1}\frac{{\left(m\rho \right)}^{j}}{j!}+\frac{{\left(m\rho \right)}^{m}}{m!(1-\rho )}\right)}^{-1}$$ (9)

And *π*_0_ is the probability of no requests waiting in the queue. Hence, *π*_*w*_ is Erlang’s C formula. We want $Pr(t\leq \hat{T})$ to be higher than a certain bound *b*, therefore by substituting Eqs (6) and (7) into Eq (5) we obtain

$$\frac{{\left(\hat{T}-(\frac{1}{\mu }+\frac{\pi_{w}}{m\mu (1-\rho )})\right)}^{2}}{(\frac{1}{\mu^{2}}+\frac{2\pi_{w}-\pi_{w}^{2}}{{\left(m\mu (1-\rho )\right)}^{2}})+{\left(\hat{T}-(\frac{1}{\mu }+\frac{\pi_{w}}{m\mu (1-\rho )})\right)}^{2}}=b$$ (10)

Eq (10) leads to a polynomial in *ρ* with maximum order 2*m* + 2. For *m* = 2, we find

$${\hat{T}}^{2}(1-b)\rho^{4}-4T_{s}^{2}b\rho^{3}-(2\hat{T}(\hat{T}-2T_{s})(1-b)-4T_{s}^{2}b)\rho^{2}+{\left(\hat{T}-2T_{s}\right)}^{2}(1-b)-4T_{s}^{2}b=0$$ (11)

We solve this equation with Newton-Raphson’s method [41]. The closer *b* is to 1, the higher is the possibility of roots being lower than 1. Once the roots of *ρ* are found, the largest positive answer is chosen, and the maximum arrival rate *λ*_*max*_ is obtained by

$$\lambda_{max}\;=\;\rho /T_{s}$$ (12)

That implies that in our algorithm, this VM is allowed to accept the coming jobs, as long as its arrival rate is not greater than *λ*_*max*_. We calculated the mean execution time (*T*_*s*_ = 1/(*mμ*)) using the moving average method [23].

$$T_{s}(i)=\alpha (execution\;time(i))+(1-\alpha ).T_{s}(i-1)$$ (13)

Where *αϵ*(0, 1) is the smoothing factor, and i is the index of the job submitted to the VM. By experiment we found that the best results are obtained when *α* = 0.2.

### 4.2. Time-shared policy

In time-shared policy, in each time slot, the VM devotes all of its processing space to the selected job. Thus, it is modeled by an M/M/1/PS queue with arrival rate *λ* and mean service time *T*_*s*_ = 1/*μ*. Therefore, the utilization factor is *ρ* = *λ*/*μ* = *λT*_*s*_. In order to find *λ*_*max*_, we find a lower limit on $Pr(t\leq \hat{T})$ as we did in space-shared policy. Hence, we use the mean and variance of the sojourn time in M/M/1/PS queue [42, 43]:

$$\mu_{t}\;=\;\frac{T_{s}}{1-\rho }$$ (14)

$$\sigma_{t}^{2}\;=\;\frac{T_{s}^{2}(2+\rho )}{{\left(1-\rho \right)}^{2}(2-\rho )}$$ (15)

Again we consider a fixed lower limit *b* for $Pr(t\leq \hat{T})$ and by substituting Eqs (14) and (15) into Eq (6), we obtain

$$\begin{array}{c} \frac{{\left(\hat{T}-\frac{T_{s}}{1-\rho }\right)}^{2}}{\frac{T_{s}^{2}(2+\rho )}{{\left(1-\rho \right)}^{2}(2-\rho )}+{\left(\hat{T}-\frac{T_{s}}{1-\rho }\right)}^{2}}=b⟹-{\hat{T}}^{2}(1-b)\rho^{3}+2\hat{T}(2\hat{T}-T_{s})(1-b)\rho^{2}-\\ ((5{\hat{T}}^{2}-6\hat{T}T_{s}+T_{s}^{2})(1-b)+T_{s}^{2}b)\rho +2({\left(\hat{T}-T_{s}\right)}^{2}(1-b)-T_{s}^{2}b)=0 \end{array}$$ (16)

Eq (17) is a third degree polynomial in *ρ*, which can be solved by Newton-Raphson’s method [41], and through its maximum positive root, we find *λ*_*max*_ by Eq (12). Moreover, *T*_*s*_ is acquired by Eq (13).

### 4.3. Hybrid mode

In the hybrid mode, in each time slot, the VM assigns each one of its cores to a different job and therefore serves more than one job in a time slot. This scenario, which is a combination of space-shared and time-shared policies, is modeled using an M/M/m/PS queue with arrival rate *λ* and mean service time *T*_*s*_ = 1/(*mμ*), and thus, the utilization factor is *ρ* = *λ*/(*mμ*) = *λT*_*s*_. Again we need the mean and variance of the sojourn time to acquire a lower limit for $Pr(t\leq \hat{T})$. Since the mean sojourn time is independent from the dispatching discipline (the queue policy for serving the customers), it is obtained by Eq (6). Braband has found a state dependent formula for waiting time of M/M/m/PS queue and derived an exact formulation for its first two moments, *M*^(1)^ and *M*^(2)^ [43]. Accordingly, for *m* = 2 the variance of the sojourn time is

$$\sigma_{t}^{2}=\frac{1}{\mu^{2}}-\frac{\rho^{2}(\rho^{2}-2\rho +2)}{\mu^{2}{\left(1-\rho^{2}\right)}^{2}}+\frac{1-\rho }{1+\rho }\left(\frac{M_{0}^{\left(2\right)}(\mu -1)}{\mu }+\frac{2M_{0}^{\left(1\right)}}{\mu (2-\rho )}\right)$$ (17)

Where $M_{0}^{\left(1\right)}$ and $M_{0}^{\left(2\right)}$ are the first and the second moments when *n*, the number of requests in the queue is zero. It has thoroughly been explained in [43] how $M_{0}^{\left(1\right)}$ and $M_{0}^{\left(2\right)}$ are acquired. For more information on this matter refer to [43, 44].

We consider a fixed lower bound *b* for $Pr(t\leq \hat{T})$ and substitute Eqs (17) and (6) into Eq (5) and find

$$\frac{{\left(\hat{T}-(\frac{1}{\mu }+\frac{\rho^{2}}{\mu (1-\rho^{2})})\right)}^{2}}{(\frac{1}{\mu^{2}}-\frac{\rho^{2}(\rho^{2}-2\rho +2)}{\mu^{2}{\left(1-\rho^{2}\right)}^{2}}+\frac{1-\rho }{1+\rho }(\frac{M_{0}^{\left(2\right)}(\mu -1)}{\mu }+\frac{2M_{0}^{\left(1\right)}}{\mu (2-\rho )}))+{\left(\hat{T}-(\frac{1}{\mu }+\frac{\rho^{2}}{\mu (1-\rho^{2})})\right)}^{2}}=b$$ (18)

Eq (18) does not lead to a polynomial in *ρ*, but a root for it can still be found by the Newton-Raphson’s method. The integrals of the $M_{0}^{\left(1\right)}$ and $M_{0}^{\left(2\right)}$ (see [43]) is solved by the Monte-Carlo method [45] in our program. Then *λ*_*max*_ is obtained from Eq (12). *T*_*s*_ is also calculated using Eq (13). Algorithm 3 describes our proposed model for finding *λ*_*max*_ in space-shared, time-shared, and hybrid policies.

**Algorithm 3**. **Determining *λ*_max_ for a VM**

Input: job_execution_time, the time the new job needs to execute;

T_s_, mean service time, calculated previously;

*W*, maximum waiting time;

root, the set of roots for the derived equation (Eq (11)) for space-shared, Eq (16)) for time-shared, and Eq (18) for hybrid policies);

Output: *λ*_*max*_

 1: *T*_*s*_ = *α*.(*job_execution_time*) + (1 − *α*).*T*_*s*_

 2: $\hat{T}=W+T_{s}$

 3: **if** *root* = = *ϕ* **then**

 4:  *λ*_*max*_ = 0

 5: **else**

 6:  max = 0

 7:  **for each** *ρ ϵ root* **do**

 8:   **if** *ρ* > *max* **then**

 9:    max = *ρ*

 10:   **end if**

 11:  **end for**

 12:  **if** *max* = = 0 **then**

 13:   *λ*_*max*_ = 0

 14:  **else**

 15:   *λ*_*max*_ = *ρ*/*T*_*s*_

 16:  **end if**

 17: **end if**

Based on experimental analysis, we found that our proposed queuing theory methods for finding the right number of jobs in a VM with space-shared, time-shared, and hybrid job scheduling policies have a small transition time and converge to steady state fast enough, so that sufficient number of VMs are activated on time.

## 5. Experimental results

**Fig 2:**
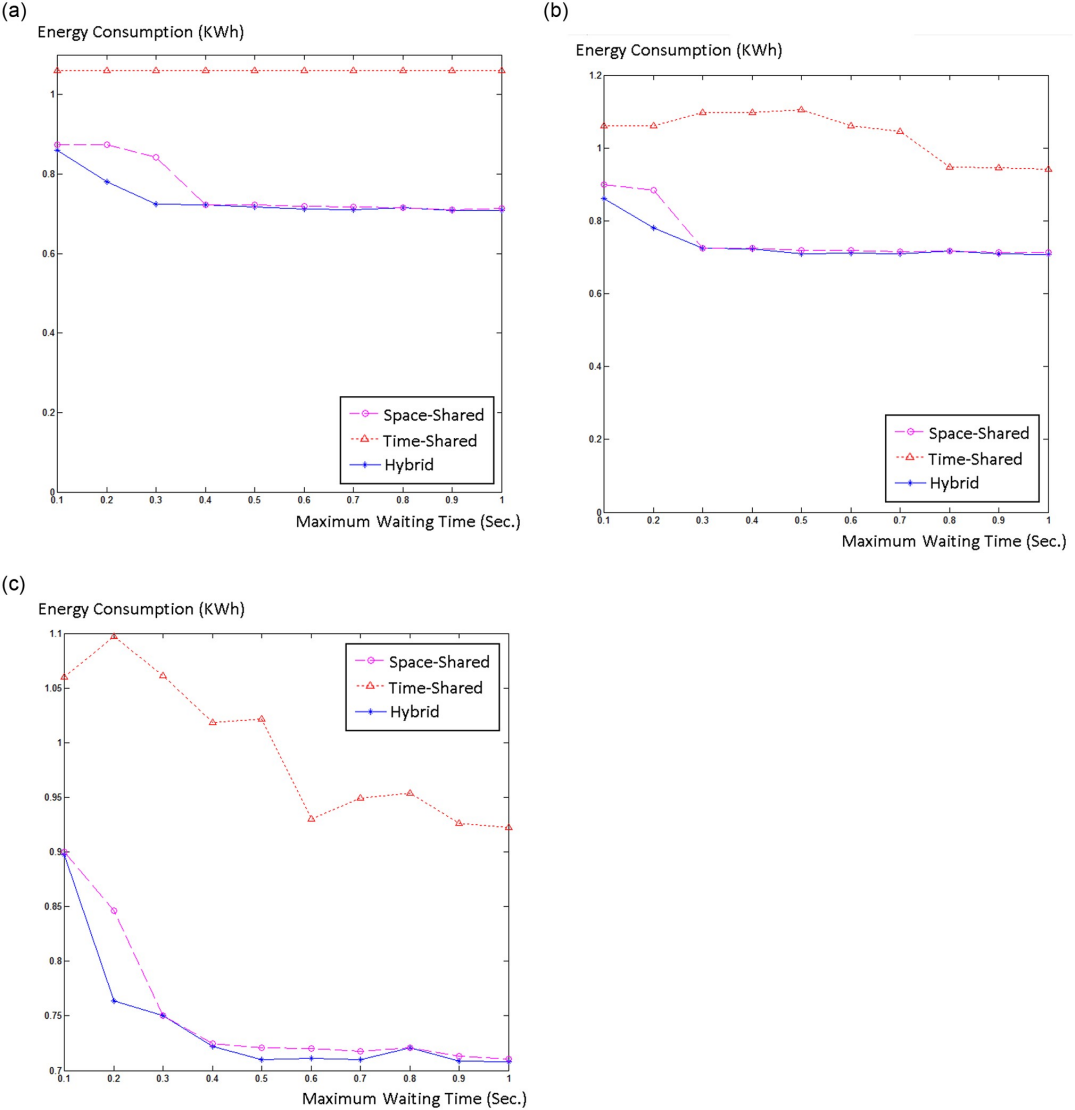
Energy consumption versus maximum waiting time for (a) b = 0.99, (b) b = 0.9, and (c) b = 0.8.

**Fig 3:**
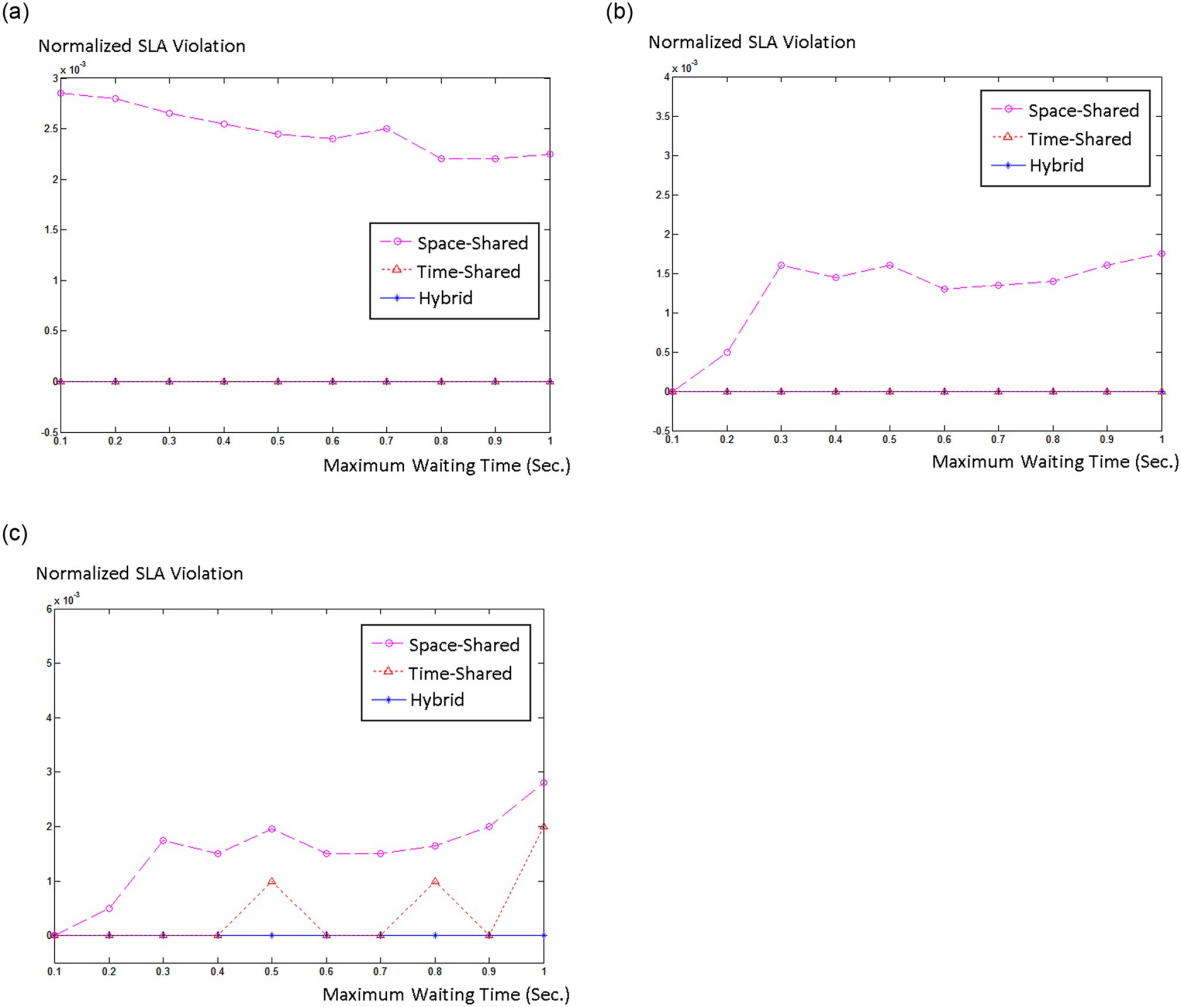
Normalized SLA violation versus maximum waiting time for (a) b = 0.99, (b) b = 0.9, and (c) b = 0.8.

To simulate and evaluate our proposed model, we interfaced CloudSim with Matlab. We used Google cluster data [46] that was collected in twenty-nine days. We created 500 hosts in CloudSim and inserted 2000 jobs into our simulation. Half of the hosts were HP ProLiant ML110 G4 (Intel Xeon 3040, 2cores×1860MHz, 4GB), and the other half were HP ProLiant ML110 G5 (Intel Xeon 3075, 2 cores×2660MHz, 4GB), as in [47]. We chose two different sizes, 500 and 800 MIPS, for the VMs. We explored the three job scheduling policies for different acceptable waiting times W. Usually, cloud users do not tolerate SLA violation, therefore we only chose higher probability bounds ($Pr(t\leq \hat{T})\geq b$), *b* = 0.99, 0.9, and 0.8 for our simulation. Fig 2 shows energy consumption versus waiting time and Fig 3 shows normalized SLA violation versus waiting time for different job scheduling policies for *b* = 0.99, 0.9, and 0.8.

It t can be seen from the diagrams that as maximum waiting time increases, energy consumption and SLA violations are both reduced. This indicates that managing real-time jobs is much harder and more complicated than non-real-time jobs. Moreover, the diagrams indicate that the hybrid policy outperforms both space-shared and time-shared policies and has lower energy consumption and fewer SLA violations compared to space-shared and time-shared policies.

**Fig 4:**
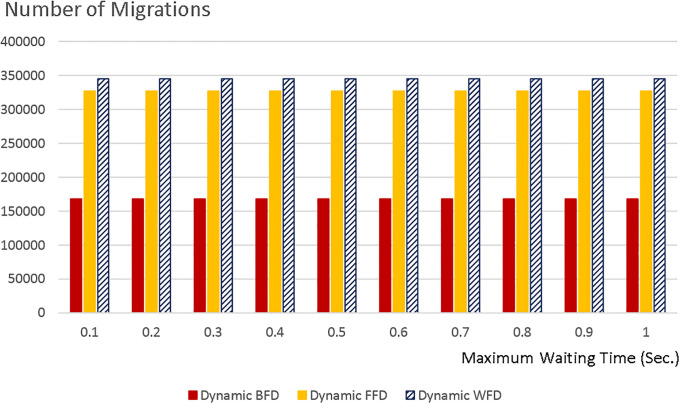
Number of VM migrations for the three dynamic algorithms for different waiting times.

**Fig 5:**
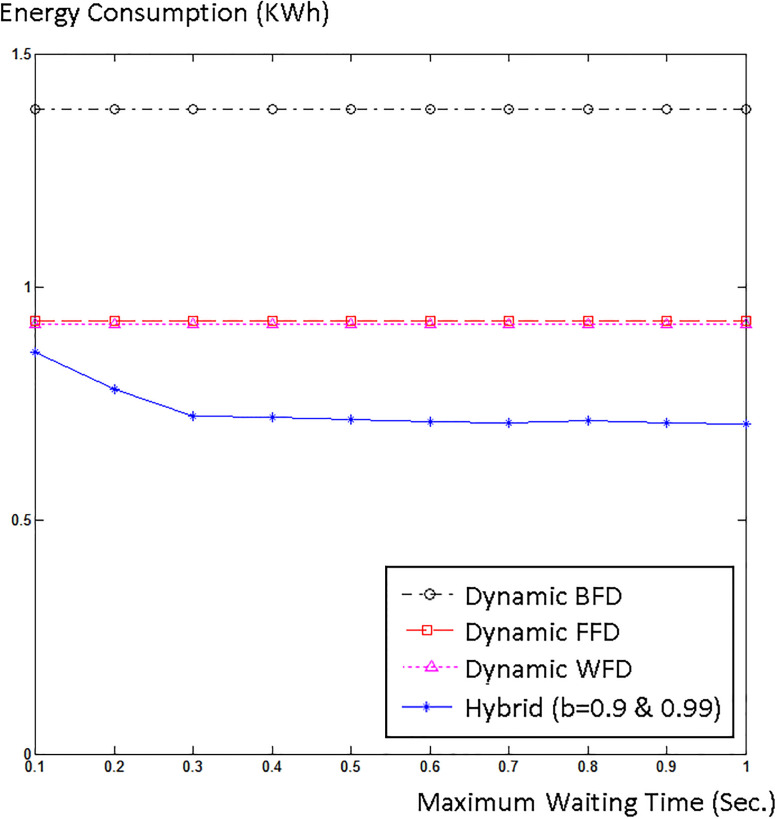
Energy consumption of our algorithm and three different VM migration algorithms.

**Fig 6:**
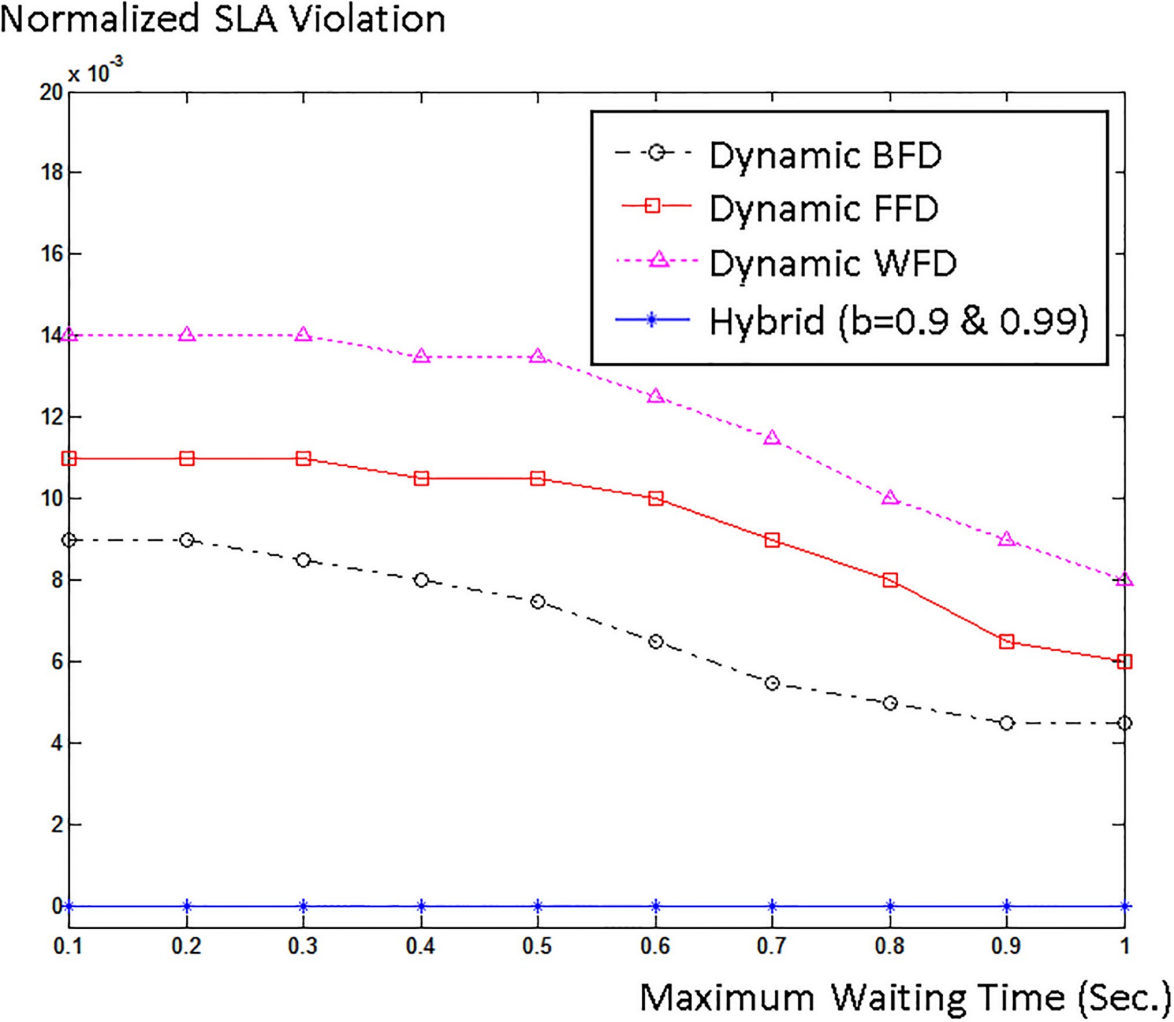
Normalized SLA violation of our algorithm and three different VM migration involved algorithms.

Next, our algorithm is compared with three different energy-aware VM placement algorithms which use VM migration. Initially, VMs are randomly placed on hosts. Every five seconds, VM placement is checked and optimized using WFD, BFD, and FFD algorithms. Fig 4 shows the number of migrations happened in each algorithm for different waiting times and Figs 5 and 6 respectively compare the energy consumption and SLA violation of each algorithm with our hybrid model for *b* = 0.99 and *b* = 0.9.

The diagrams show that our algorithm outperforms WFD, BFD, and FFD algorithms with VM migration. The reason is that our algorithm places VMs on physical machines in a WFD fashion without migration. VM migration prolongs the execution process and raises the probability of SLA violation. Even in live migration the jobs have to stop and wait until their essential codes and data stacks are moved, and then continue their execution. Moreover, VM migration introduces a burst to the network and causes even more latency. Thus, especially for real-time applications, VM migration brings about SLA violation. In addition, some additional amount of energy is consumed by the network to migrate the VMs. Therefore, the energy consumption and SLA violation of the three algorithms with VM migration degrades and our energy-aware non-migration VM placement excels them. Corresponding to any SLA violation level, the energy consumption of our method is better than that of dynamic FFD, BFD, and WFD methods. So, our hybrid algorithm presents lower energy consumptions and fewer SLA violations and outperforms the three dynamic algorithms. We also found that the energy consumption of the three dynamic algorithms is highly affected by the number of migrations. Therefore, since the number of migrations of the three dynamic algorithms does not noticeably change as the waiting time rises, in Fig 5, the energy consumption of them does not decrease; however, as waiting time rises less jobs elapse their deadlines and fewer SLA violations occur, according to Fig 6.

## 6. Conclusion

In this paper, an energy-aware non-migration VM placement and hybrid job scheduling policy was proposed to make the cloud elastic. Generally, job scheduling policies can be classified into two main groups, namely, space-shared and time-shared. Moreover, some algorithms, which we call hybrid, combine the two aforementioned algorithms. Proposed methods which completely avoid VM migration, usually require the workload to be predictable. However, in our approach, the workload do not necessarily need to be predictable. In addition, our method is applicable for both real-time and non-real-time applications. We showed in our paper that hybrid policy excels space-shared and time-shared policies in terms of reducing both energy consumption and SLA violation. We adjusted the resources allocated to each cloud user according to their QoS by modeling the VMs of a data center with a queuing theory technic. We applied our idea to space-shared, time-shared, and hybrid policies, developed different equations for each one, and obtained the maximum acceptable arrival rates *λ*_*max*_ by studying the sojourn time of the jobs. *λ*_*max*_ was used to determine whether a VM can accept more jobs or not. An energy-aware non-migration algorithm was designed to place the VMs on servers in a WFD fashion.

We interfaced CloudSim with Matlab to simulate and evaluated our algorithm. The results show that as waiting time increases, both energy consumption and SLA violation decrease. This clearly shows that although real-time jobs are not minded in previous research deservedly, managing real-time jobs are much more complicated than non-real-time jobs. The experimental results indicate that the hybrid policy is superior to space-shared and time-shared policies in reducing both energy consumption and SLA violation. Moreover, we compared hybrid algorithm with three different algorithms, WFD, BFD, FFD with migration. The results show that our energy-aware non-migration VM placement algorithm with hybrid job scheduling policy outperforms all three in terms of reducing both energy consumption and SLA violation.

## Supporting information

S1 Data (ZIP)
