# Supplementary material for: A static VM placement and hybrid job scheduling model for green data centers
Source: PLoS One. 2020 Aug 13;15(8):e0237238. doi: 10.1371/journal.pone.0237238 (PMC7425884; doi:10.1371/journal.pone.0237238)
Supplement: S1 Data — (ZIP) [file pone.0237238.s001.zip › MyProject/dataset/Google cluster-usage traces format schema.pdf]

Google cluster-usage traces: format + schema

Charles Reiss, John Wilkes, Joseph Hellerstein
Version of 2013-05-06, for trace version 2. Revised 2014-11-17 for trace version 2.1.
Status: exported outside Google.
Copyright © 2011 Google Inc. All rights reserved.

Table of contents

- Introduction
- Common techniques and fields
  - Obfuscation techniques
  - Time and timestamps
  - Unique identifiers
  - User and job names
  - Resource units
- Data tables
  - Machines
    - Machine events
    - Machine attributes
  - Jobs and tasks
    - Job and task life cycles and event types
    - Missing event records
    - Job events table
    - Task events table
  - Task constraints
  - Resource usage
- File format
- Downloading the data
- Anomalies
  - Data collection
  - Missing information
- Appendices
  - Document history

Introduction

This document describes the semantics, data format, and schema of usage traces of a Google compute cell. This document describes version 2.1 of the trace format, and should be read in conjunction with the detailed information provided with a trace.

A Google *cluster* is a set of machines, packed into racks, and connected by a high-bandwidth cluster network. A *cell* is a set of machines, typically all in a single cluster, that share a common cluster-management management system that allocates work to machines. Work arrives at a cell in the form of *jobs*. A job is comprised of one or more *tasks*, each of which is accompanied by a set of resource requirements used for scheduling (packing) the tasks onto machines. Each task represents a Linux program, possibly consisting of multiple processes, to be run on a single machine. Tasks and jobs are scheduled onto machines according to the lifecycle described below. Resource requirements and usage data for tasks are derived from information provided by the cell's management system and the individual machines in the cell.

A single usage trace typically describes several days of the workload on one of these compute cells. A trace is made up of several *datasets*. A dataset contains a single *table*, indexed by a

primary key that typically includes a timestamp. Each dataset is packaged as a set of one or more files, each provided in a compressed CSV format. (See below for the details.)

One of our goals in publishing this trace data is to make visible many of the scheduling complexities that affect Google's workload, including the variety of job types, complex scheduling constraints on some jobs, mixed hardware types, and user mis-estimation of resource consumption. We hope that researchers will find it useful.

Common techniques and fields

This section describes the representation and semantics of fields that occur in several tables. Note that a trace may not include all the types of data described here.

Obfuscation techniques

For confidentiality reasons, we have obfuscated certain information in the trace. In particular, most free-text fields have been randomly hashed, resource sizes have been linearly transformed (scaled), and certain values have been mapped onto a sorted series. We took care to do this in a consistent way so that the data can still be used in research studies.

Here are the obfuscation transformations that are used for most data types:

- *unchanged*: the values are not obfuscated.
- *hashed*: each value is transformed to an opaque value by means of a keyed cryptographic hash.
- *ordered*: the list of observed (or possible) values is generated and sorted. Then, the items in this list are assigned sequential numbers starting with 0 (i.e., gaps are elided), and the observed values are mapped onto these numbers before adding them to the trace.
- *rescaled*: each value is transformed by dividing by the maximum value that appears anywhere in the trace for that measurement. For example, the maximum observed memory capacity across all machines is used to rescale all memory requests and usage values. (The maximum is used to guarantee no dividing by zero and that no extra information leaks into the result.) This value is then rounded to a granularity of $max(1.0/2^{10}, max\_value/2^{20})$ - i.e., no more than ten bits of binary precision or ~1 millionth of the maximum value, whichever is larger. Almost all floating point values are transformed in this way.
- *special*: a few values are treated in a special way.

Time and timestamps

Each record has a timestamp, which is in microseconds since 600 seconds before the beginning of the trace period, and recorded as a 64 bit integer (i.e., an event 20 seconds after the start of the trace would have a timestamp=620s).

Additionally, there are two special time values representing events that did not occur within the trace window:

- A time of 0 represents events that occurred before the beginning of the trace window. Examples: machines that existed at the start of the trace; a task or job that was submitted (or has already been scheduled) before start of the the trace.
- A time of 2^{63}-1 (MAXINT) represents events that occur after the end of the trace window (e.g., if there is a data collection failure near the end of the trace window).

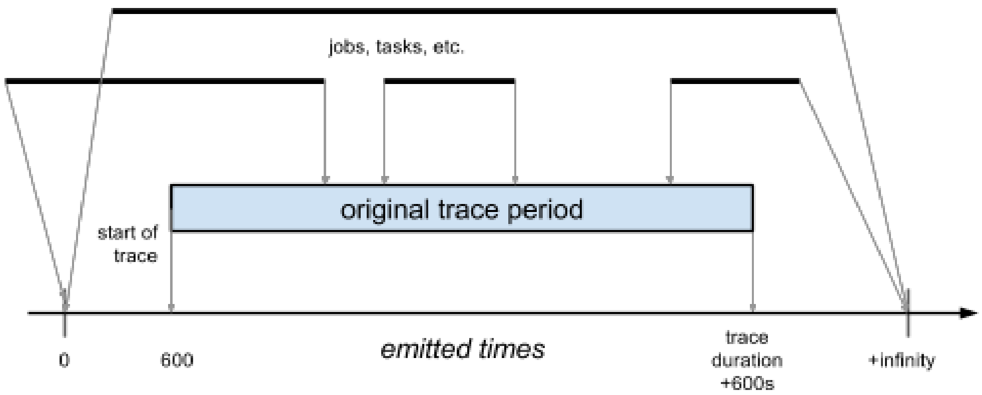

Mapping of original times to times emitted in the trace.

As an exception, times in usage measurements are treated slightly differently, because the the maximum measurement length is 300 seconds. We apply the same start-time offset to these times as we do for events inside the trace window; this is enough to ensure a clear separation between events before the trace and other events. Note that a measurement interval may overlap the beginning or end of the trace. Additionally, the best available precision for usage measurements is to the nearest second; we still report these times in microseconds for consistency.

Timestamps are approximate, although reasonably accurate. They are sourced from multiple machines, and so small disagreements in timestamps may reflect clock drift between machines in the cluster instead of any real scheduling or utilization issue.

Unique identifiers

Every job and every machine is assigned a unique 64-bit identifier. These IDs are never reused; however, machine IDs may stay the same when a machine is removed from the cluster and returned. Very rarely, job IDs may stay the same when the same job is stopped, reconfigured and restarted. (We are aware of only one such example in our first trace. Usually, restarting a job will generate a new job ID but keep the same job name and user name.)

Tasks are identified by the job ID of their job and a 0-based index within the job. Tasks with the same job ID and task index can be (and frequently are) stopped and be restarted without assigning a new job ID or index.

User and job names

User and job names are hashed and provided as opaque base64-encoded strings that can be tested for equality.

The *logical job name* is a heuristically normalized name of the job that combines data from several internal name fields, and hashes the result to construct an opaque base64-encoded string (e.g., most numbers in the logical name are replaced with a fixed string). In cases where unique job names are generated to avoid conflicts, job names generated by different executions of the same program will usually have the same logical name. MapReduce is an example of a Google system that frequently generates unique job names.

Usernames in this trace represent Google engineers and services. Production jobs run by the same username are likely to be part of the same external or internal service. Whenever a

single program runs multiple jobs (for example, a MapReduce program spawns both a master job and worker job), those jobs will almost always run as the same user.

Resource units

Most resource utilization measurements and requests have been normalized, including:

- CPU (core count or core-seconds/second)
- memory (bytes)
- disk space (bytes)
- disk time fraction (I/O seconds/second)

For each of the foregoing, we compute separate normalizations. The normalization is a scaling relative to the largest capacity of the resource on any machine in the trace (which is 1.0). This is true even for disk space and disk time fraction for which we do not provide individual machine capacities in this version.

Data tables

Below we describe the tables that will be provided. Table keys are *italicized*; note that some keys are constructed from multiple fields. Reminder: not all traces will include all the types of data described here. **The columns might occur in a different order, or have different names than reported here: the definitive specification of such details can be found in the `schema.csv` file.**

Machines

Machines are described by two tables: the *machine events table* and the *machine attributes table*.

Machine events

Each machine is described by one or more records in the machine event table. The majority of records describe machines that existed at the start of the trace.

Machine events table:

1. *timestamp*
2. *machine ID*
3. event type
4. platform ID
5. capacity: CPU
6. capacity: memory

There are three types of machine events:

- ADD (0): a machine became available to the cluster - all machines in the trace will have an ADD event.
- REMOVE (1): a machine was removed from the cluster. Removals can occur due to failures or maintenance.
- UPDATE (2): a machine available to the cluster had its available resources changed.

Machine capacities reflect the normalized physical capacity of each machine along each dimension. Each dimension (CPU cores, RAM size) is normalized independently. Note that not all of this capacity is available to tasks; for example, the cluster scheduler needs to reserve some resources for itself and the operating system.

The platform ID is an opaque string representing the microarchitecture and chipset version of the machine. Different combinations of micro-architectures (e.g. vendor chip code-names like Nehalem or Bulldozer) and memory technologies (e.g. DDR2 versus DDR) will result in different platform IDs. Two machines with the same platform ID may have substantially different clock rates, memory speeds, core counts, etc.

Machine attributes

Machine attributes table:

1. *timestamp*
2. *machine ID*
3. *attribute name:* an opaque string
4. attribute value: either an opaque string or an integer
5. attribute deleted: a boolean indicating whether the attribute was deleted

Machine attributes are key-value pairs representing machine properties, such as kernel version, clock speed, presence of an external IP address. Tasks can specify constraints on machine attributes.

Machine attribute values are provided as integers if there are a small number of values for that attribute name or as obscured strings otherwise. In the first case, the values of the attributes observed across all machines are recorded and sorted (in numerical order, if all are numbers). The first value is mapped to 1, the second to 2, and so on. This strategy is the same as used in a prior Google publication of some related data.[1] Some attributes have no associated values because their presence or absence is the signal (e.g., whether a machine runs a GFS chunkserver); these attributes are given the value 1 if present.

Jobs and tasks

Jobs and tasks are described by these tables:

- Job events table
- Task events table
- Task constraints table

The two event tables describe jobs/tasks and their lifecycles. The constraints table describes task placement constraints that restrict the machines onto which tasks can schedule.

Usually all tasks within a job execute exactly the same binary with the same options and resource request. Programs that need to run different types of tasks with different resource requirements usually execute as multiple jobs. For example, MapReduce runs masters and workers in separate jobs.

Job and task life cycles and event types

[1] "Modeling and Synthesizing Task Placement Constraints in Google Compute Clusters", Victor Chudnovsky, Rasekh Rifaat, Joseph Hellerstein, Bikash Sharma, Chita Das, Symposium on Cloud Computing (SoCC), Oct. 2011.

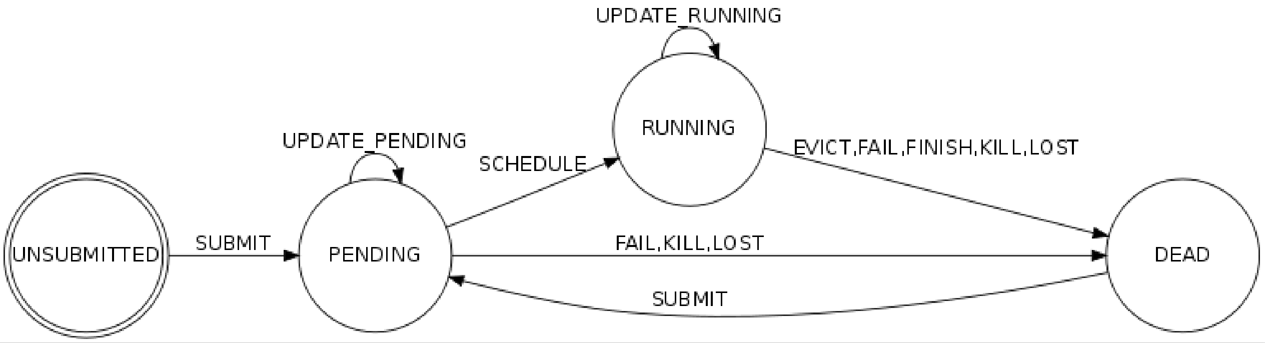

State transitions for jobs and tasks.

The diagram above shows a simplified model of the states through which a job or task progresses. Job and task events indicate transitions between these states.

There are basically two types of events: ones that affect the scheduling state (e.g., a job is submitted, or it gets scheduled and becomes runnable, or its resource requests are updated), and ones that reflect state changes of a task (e.g., the task exits).

Each job and task event has a value representing the type of event. The state of the job or task after the event can always be determined from this event type. For job or task deaths, the event type also contains information about the cause of the death. Here are the event (transition) codes:

- SUBMIT (0): A task or job became eligible for scheduling.
- SCHEDULE (1): A job or task was scheduled on a machine. (It may not start running immediately due to code-shipping time, etc.) For jobs, this occurs the first time *any* task of the job is scheduled on a machine.
- EVICT(2): A task or job was descheduled because of a higher priority task or job, because the scheduler overcommitted and the actual demand exceeded the machine capacity, because the machine on which it was running became unusable (e.g. taken offline for repairs), or because a disk holding the task's data was lost.
- FAIL(3): A task or job was descheduled (or, in rare cases, ceased to be eligible for scheduling while it was pending) due to a task failure.
- FINISH(4): A task or job completed normally.
- KILL(5): A task or job was cancelled by the user or a driver program or because another job or task on which this job was dependent died.
- LOST(6): A task or job was presumably terminated, but a record indicating its termination was missing from our source data.
- UPDATE_PENDING(7): A task or job's scheduling class, resource requirements, or constraints were updated while it was waiting to be scheduled.
- UPDATE_RUNNING(8): A task or job's scheduling class, resource requirements, or constraints were updated while it was scheduled**.**

The simplest case is shown by the top path in the diagram above: a job is SUBMITted and gets put into a pending queue; soon afterwards, it is SCHEDULEd onto a machine and starts running; some time later it FINISHes successfully.

If a task or job that has just EVICTed, FAILed, or KILLed remains runnable, a SUBMIT event will appear immediately after the descheduling events. (I.e., the system tries to restart jobs and tasks that have failed.) The actual policy about how many times tasks can be rescheduled in spite of non-normal termination varies between jobs. For example, though rare, it is

possible for some tasks to be indefinitely descheduled after an EVICT event that was caused by the scheduler not giving the task its requested memory.

The trace may contain both SUBMIT and SCHEDULE events for the exact same microsecond for a job or task. This means that a job or task was submitted and immediately scheduled, or, if the timestamp on the events is 0, that the job or task was submitted and scheduled before the trace began.

Unfortunately, we are not able to precisely determine the cause of all task deaths; where we are uncertain, we map this to a KILL event, which also includes cases where an external program or a developer took explicit action to terminate it. There is no information about whether a job or task was executing normally in either case.

Note that this is a simplified version of the scheduling system we actually employ, and a few of the more sophisticated features have been been mapped down into this model. We do not believe that any significant fidelity has been lost in this process from the point of view of resource-scheduling research.

Missing event records

Our data sources contain both time-series of job and task changes and periodic snapshots of job and task state, so we are able to check for consistency between them. When it seems we are missing an event record, we synthesize a replacement. Similarly, we look for a record of every job or task that is active at the end of the trace time window, and synthesize a missing record if we don't find one.

Synthesized records have a number (called the "missing info" field) to represent why they were added to the trace:

- SNAPSHOT_BUT_NO_TRANSITION (0): we did not find a record representing the given event, but a later snapshot of the job or task state indicated that the transition must have occurred. The timestamp of the synthesized event is the timestamp of the snapshot.
- NO_SNAPSHOT_OR_TRANSITION (1): we did not find a record representing the given termination event, but the job or task disappeared from later snapshots of cluster states, so it must have been terminated. The timestamp of the synthesized event is a pessimistic upper bound on its actual termination time assuming it could have legitimately been missing from one snapshot.
- EXISTS_BUT_NO_CREATION (2): we did not find a record representing the creation of the given task or job. In this case, we may be missing metadata (job name, resource requests, etc.) about the job or task and we may have placed SCHEDULE or SUBMIT events latter than they actually are.

Records without missing data have no value (empty string) in the "missing info" field.

Job events table

The job events table contains the following fields:

1. *timestamp*
2. missing info
3. *job ID*
4. event type
5. user name
6. scheduling class
7. job name
8. logical job name

The job/task event tables include any jobs that are active (RUNNING) or eligible to run but waiting to be scheduled (PENDING) at any point in the trace. For every job in the trace, we will include at least one record for all its tasks, which will include its scheduling constraints.

All jobs and tasks have a *scheduling class* that roughly represents how latency-sensitive it is. The scheduling class is represented by a single number, with 3 representing a more latency-sensitive task (e.g., serving revenue-generating user requests) and 0 representing a non-production task (e.g., development, non-business-critical analyses, etc.). Note that scheduling class is *not* a priority, although more latency-sensitive tasks tend to have higher task priorities. Scheduling class affects machine-local policy for resource access. Priority determines whether a task is scheduled on a machine.

The *job name* is hashed and provided as opaque base64-encoded strings that can be tested for equality. Unique job names are sometimes generated by automated systems to avoid conflicts. (MapReduce is an example of a Google system that does this.)

The *logical job name* is an opaque name that combines data from several internal name fields (e.g., most numbers in the logical name are replaced with a fixed string). Logical job names partially compensate for the unique job names generated by automatic tools; in this case, different executions of the same program will usually have the same logical name.

Task events table

The task events table contains the following fields:

1. *timestamp*
2. missing info
3. *job ID*
4. *task index* - within the job
5. machine ID
6. event type
7. user name
8. scheduling class
9. priority
10. resource request for CPU cores
11. resource request for RAM
12. resource request for local disk space
13. different-machine constraint

The machineID, if present, indicates the machine onto which the task was scheduled.

Each task has a *priority,* a small integer that is mapped here into a sorted set of values, with 0 as the lowest priority (least important). Tasks with larger priority numbers generally get preference for resources over tasks with smaller priority numbers.

There are some special priority ranges:

- "free" priorities: these are the lowest priorities. Resources requested at these priorities incur little internal charging.
- "production" priorities: these are the highest priorities. The cluster scheduler attempts to prevent latency-sensitive tasks at these priorities from being evicted due to over-allocation of machine resources.
- "monitoring" priorities: these priorities are intended for jobs which monitor the health of other, lower-priority jobs

We may indicate which priority ranges represent which categories in the documentation for

each trace; since we normalize the provided priorities, these ranges may differ between releases.

If the *different-machine constraint* field is present, and true, it indicates that a task must be scheduled to execute on a different machine than any other currently running task in the job. It is a special type of constraint.

The resource requests represent the maximum amount of CPU, memory, or disk space a task is permitted to use (we call this its *limit*). Tasks using more than their limit may be throttled (for resources like CPU) or killed (for resources like memory). Note that the scheduler may over-commit resources on a machine. As a result, it can happen that there are not enough resources to meet all the runtime requests from the tasks, even though each of them is using less than its limit. If this happens, one or more low priority task(s) may be killed.

Additionally, the runtime environment sometimes permits tasks to use more than their request. For example, tasks are permitted to use free CPU capacity on the machine, so tasks with brief latency-insensitive CPU bursts might usefully run with a requested CPU allocation of 0.

This trace includes some jobs that support a Google distributed filesystem, but which do *not* request disk space for the file system's persistent storage via the cluster scheduler - they rely on an external allocation mechanism. These resource requests and usage are not included in the trace data.

Task constraints

A task may have zero or more task placement constraints, which restrict the machines on which the task can run. Each constraint record corresponds to exactly one task event record. (If a task is updated only by updating the machine constraints, then there will be a task event record indicating an update.) There may be multiple constraints on a single attribute, for example an attribute may have both a less than and a greater than constraint for a single task.

The task constraints table contains the following fields:

1. *timestamp*
2. *job ID*
3. *task index*
4. *attribute name* -- corresponds to machine attribute table
5. attribute value -- either an opaque string or an integer or the empty string
6. *comparison operator*

The comparison operators are:

- LESS THAN (2), GREATER THAN (3): Represent the machine attribute as an integer (or 0 if the attribute is not present), then compare it to the supplied attribute value. These comparisons are *strictly less than* and *strictly greater than*;
- EQUAL (0), NOT EQUAL (1): Represent the machine attribute as a string (or as the empty string if it is not present), then compare it to the supplied attribute value;

Resource usage

Our clusters uses Linux containers for resource isolation and usage accounting. Each task runs within its own container and may create multiple processes in that container.

We report usage values from each measurement period, which is typically 5 minutes (300s), although some measurement records span a shorter time, typically because the task is updated within that time period. When a task is killed, the measurement time period may extend for up to tens of seconds beyond when the task was terminated, and a few measurement records extend a few minutes after the time the task was terminated.

Within each measurement period, measurements are usually taken at 1 second intervals, although system load sometimes prevents the resource information from being sampled at the desired rate, so a measurement may span more than 1 second. To assist in identifying these cases, we provide the *sample portion* (aka *sampling rate*) which is the ratio between the number of expected samples (e.g., 300 for a 300 s measurement window) to the number of observed samples.

The 1 second samples are aggregated over the length of each measurement period to provide a mean value for the measurement period as well as the maximum value seen in any 1 second sample in that period.

In some cases, the aggregate value for the container is constructed from measurements obtained from multiple sub-containers: although we know the maximum usage for each subcontainer, our measurement system may not report the true maximum of the outer container. In this case, the maximum value we report is the sum of the maximum values from the subcontainers, and records with maximum usage aggregated in this manner will be marked with an *aggregation type* marker of 1; all other records will have an aggregation type of 0. Additionally, we may not have maximum values for all of the sub-containers (e.g., if they are small and/or short-lived), even though the average usage measurement includes their usage.

Measurement records may be missing. Missing records do not necessarily indicate that a task was not running.

There are some task usage measurements for periods when no process belonging to the task was running in the task's container. For example, these measurements might occur while binaries are being copied to the machine. In this case, memory, CPU, and disk usage for a task may legitimately be 0. In some cases, a task may run with no process for an extended period of time.

The task resource usage table contains these fields:

1. *start time* of the measurement period
2. *end time* of the measurement period
3. *job ID*
4. *task index*
5. machine ID
6. mean CPU usage rate
7. canonical memory usage
8. assigned memory usage
9. unmapped page cache memory usage
10. total page cache memory usage
11. maximum memory usage
12. mean disk I/O time
13. mean local disk space used
14. maximum CPU usage
15. maximum disk IO time
16. cycles per instruction (CPI)
17. memory accesses per instruction (MAI)

18. sample portion
19. aggregation type (1 if maximums from subcontainers were summed)
20. sampled CPU usage: mean CPU usage during a random 1s sample in the measurement period *(only in v2.1 and later)*

CPU usage (also known as CPU rate) is measured in units of CPU-core seconds per second: if a task is using two cores all the time, it will be reflected as a usage of 2.0 core-s/s.

- The "CPU usage - sampled" field is only provided in version 2.1 and later. It is the average CPU usage during a one-second period that is picked uniformly at random from the 5-minute measurement period used for the other data in the row. For long-running tasks, this data can be used to build a reasonably accurate stochastic model of CPU usage.

Since memory isolation is achieved through Linux containers, some kernel memory usage on behalf of the task is accounted to the task. Tasks must request sufficient memory to include such allocations. The following memory usage data are included:

- memory usage: canonical memory usage measurement; the number of user accessible pages, including page cache but excluding some pages marked as stale.
- unmapped page cache memory: Linux page cache (file-backed memory) not mapped into any userspace process;
- page cache memory: total Linux page cache (file-backed memory)
- assigned memory: memory usage based on the memory actually assigned to the container (but not necessarily used)
- maximum memory usage: the maximum value of the canonical memory usage measurement observed over the measurement interval. This value is not available for some tasks.

Disk I/O time is measured using the *blkio* subsystem for Linux containers. Usage measurements are the sum across all disks on the machine, in units of disk-time seconds per second.

Disk space recorded in this trace represents runtime local disk capacity usage. Disk usage required for binaries and other read-only, pre-staged runtime files is *not* included. Additionally, most disk space used by distributed, persistent storage (e.g. GFS, Colossus) is not accounted for in this trace.

CPI and MAI statistics are collected from processor performance counters; not all machines collect this data. Memory accesses are based on measurements of last-level cache misses.

File format

A trace is a set of datasets, one each for the different tables described above. Each dataset is made up of one or more files, each of which is compressed using gzip.

Each file is in CSV format, using Unix-style line endings (ASCII LF). The CSV files have no header. Rows are sorted by the event timestamp, which is always stored in the first column.

The filenames used match the following patterns (`?????` represents a zero-padded five-digit number, e.g., 00123).

```
README
MD5SUM
SHA1SUM
```

```
SHA256SUM
schema.csv
job_events/part-?????-of-?????.csv.gz
task_events/part-?????-of-?????.csv.gz
machine_events/part-?????-of-?????.csv.gz
machine_attributes/part-?????-of-?????.csv.gz
task_constraints/part-?????-of-?????.csv.gz
task_usage/part-?????-of-?????.csv.gz
```

Field formats are as follows:

1. If there is no value for a field, then the field will be empty (no quotes).
2. Numerical fields are represented as decimal integers (for timestamps, IDs, event types, etc.) or floating point values (for resource utilization numbers) as might be printed out using the C printf format "%g".
3. Boolean fields are represented as 0 (false) or 1 (true).
4. String fields are base-64 encoded values of SHA256 hashes and so will have constant length.

All fields have been normalized so they do not contain commas, newlines or quotes. This means that each line of the CSV file should be parsable by splitting on commas.

Large tables are split into several consecutive files, numbered in order by time. CSV records are never split across files, but the records for a particular job, task, or machine may be split across files. The same timestamp ranges will be used for all files with the same file number across the different datasets (e.g., `task_events-01234-of-05678.csv.gz` will contain the same range of timestamps as `machine_events-01234-of-05678.csv.gz`).

Downloading the data

The data is stored in Google Storage for Developers. Each trace uses its own bucket. (As an example in what follows, we will use the v2.1 trace from 2011, which is called clusterdata-2011-2.) You do not need to have a Google account or sign up for Google Storage to download the data.

Included with each trace is a SHA256SUM file, which can be used to verify the integrity of a download, using the sha256sum command from GNU coreutils using a command like

```
sha256sum --check SHA256SUM
```

There are three ways to download the data: with the GSUtil utilities (this is the recommended approach); via the boto library, or simply using HTTP. Note that an entire trace is likely to be moderately large (a few tens of GB); see the individual trace descriptions for more information.

Command line: GSUtil

First, download the GSUtil software. Then you use a command like

```
gsutil ls gs://clusterdata-2011-2/
```

to list the contents of the bucket and a command like

```
gsutil cp -R gs://clusterdata-2011-2/  destination-directory
```

to copy the contents of the bucket to your local disk. You can also select individual sets of files

within the bucket using directory names or pattern matches like "`gs://clusterdata-2011-2/task_usage/*`". If you have a fast connection, you may find it helpful to use the `-m` option to download multiple files in parallel. If your download is interrupted, gsutil will resume that download if you attempt to download on top of the partially-transferred files.

Programmatically: boto library
You can download the data from Google Storage programmatically using the boto library.

Web: HTTP
Additionally, each file in Google Storage is available using HTTP. For example, `https://commondatastorage.googleapis.com/clusterdata-2011-2/SHA256SUM` is the URL for the SHA256SUM file for the first trace. Unfortunately, there are no directory indices.

Anomalies

This section lists a few oddities you should know about.

Data collection

This data is primarily derived from monitoring data, collected by periodic remote procedure calls (RPCs). When the monitoring system or cluster gets overloaded, data may not be collected. Additionally, we have deliberately removed some usage data which appears to have been mislabeled due to a bug in the monitoring system. For scheduler events, we have synthesized records to make the data consistent as described above, but some data is still likely to be missing from the trace.

See the detailed description that comes with a trace to see how many event records and resource usage measurements it is missing.

Missing information

Our cluster scheduler supports several features that are not represented in the traces. These include:

- Inter-job dependencies: users can submit jobs specifying that they cannot be run until another job finishes or can only run while another job is running. Our traces show jobs as submitted when any such job dependencies are satisfied. Jobs whose dependencies are never satisfied are not present. Jobs or tasks killed because a related job dies are shown as a KILL event.

- Other resources: some additional resource types and their usage.

- Other constraints: some non-resource scheduling constraints (for example, limiting the number of tasks within a job that can be on the same rack); "preferred" constraints, where the task will request to run on a machine with a particular attribute but still be willing to schedule elsewhere.

- Workload not managed by the scheduler: the task usage and machine capacity measurements do not account for some of the workload on the machines, because some systems run outside the control of the cluster scheduler.

Appendices

Document history (external)

Date	*Notes*
2014-11-17	Added info about cluster-2011-2.
2014-09-02	Version 2.1: added "CPU usage (aka rate) - random 1s sample" to task-usage table
2013.05.06	Added page numbers; highlighted text pointing out that schema.csv is the definitive reference for columns; explained that CPU usage and CPU rate are the same thing.
2012.03.20	Added note about column order being defined by the schema.csv file; fixed the task-constraint table to match the data.
2011.11.08	Added note about gsutil “max open files” restriction.
2011.10.27	First externally-published version.
