# Supplementary material for: A static VM placement and hybrid job scheduling model for green data centers
Source: PLoS One. 2020 Aug 13;15(8):e0237238. doi: 10.1371/journal.pone.0237238 (PMC7425884; doi:10.1371/journal.pone.0237238)
Supplement: S1 Data — (ZIP) [file pone.0237238.s001.zip › MyProject/dataset/ReadMe.docx]

Dear Reader,

We have used Google Cluster Data as our dataset. It can be downloaded form here:

<https://commondatastorage.googleapis.com/clusterdata-2011-2/>

It has a very useful user manual that has explained all the files in detail. We have included the user manual in the MyProject\dataset folder (MyProject\dataset\Google cluster-usage traces format schema 2014-11-17 external.pdf)

We have extracted the jobs from the first 6 files and placed them in dataset0.csv to dataset6.csv.

The program for extracting the jobs can be found at “buildDataset.java”.
