# Supplementary material for: A static VM placement and hybrid job scheduling model for green data centers
Source: PLoS One. 2020 Aug 13;15(8):e0237238. doi: 10.1371/journal.pone.0237238 (PMC7425884; doi:10.1371/journal.pone.0237238)
Supplement: S1 Data — (ZIP) [file pone.0237238.s001.zip › MyProject/Minimal Dataset.docx]

In this document we explain how you can reach to the results and conclusions of our paper, “A static VM placement and hybrid job scheduling model for green data centers”.

In the “MyProject” folder you find 3 different folders; namely, “dataset”, “withMatlab”, and “withoutMatlab”. Here, we explain what each folder includes:

- dataset: this folder includes the first 6 task-event files of google cluster data (“task_events-part-00000-of-00500.csv”, “task_events-part-00001-of-00500.csv”, “task_events-part-00002-of-00500.csv”, “task_events-part-00003-of-00500.csv”, “task_events-part-00004-of-00500.csv”, and “task_events-part-00005-of-00500.csv”).

We have extracted the jobs of these files (“dataset0.csv”, dataset1.csv”, “dataset2.csv”, “dataset3.csv”, “dataset4.csv”, and “dataset5.csv”).

The code for extracting the “dataset*.csv” files from “task_events-part-0000*-of-00500.csv” is provided in “buildDataset.java”.

More information can be found in the user manual of Google cluster data which you can find in “Google cluster-usage traces format schema.pdf” file.

The results in our manuscript is obtained using “dataset2.csv”.

- withMatlab: as we have reported in our manuscript, we have interfaced Matlab with CloudSim in our simulation. The code is available in the “withMatlab” folder. Executing our code with Matlab requires a great amount of processor capacity and memory space. If you need a lighter code, you can execute our code without Matlab, which is available in “withoutMatlab” folder.
- withoutMalab: the code in this folder is much lighter than the code in the “withMatlab” folder. If you were not able to execute our code in the “withMatlab” folder because it required more CPU capacity and memory space, you can use the code available in the “withoutMatlab” folder. We have gained the results in our manuscript using the code in the withoutMatlab folder, but one should be able to get the same results using the code in the withMatlab folder.

In each of the “withMatlab” and “withoutMatlab” folders, we have included a “ReadMe.docx” file which explains how to execute the codes. Additionally, in each of these folders there are 6 different folders; namely, “TimeShare”, “SpaceShare”, “Hybrid”, “dynamicWfd”, “dynamicFfd”, and “dynamicBfd”. These 6 folders correspond to the different parts of our research presented in our manuscript (time-share policy, space-share policy, hybrid policy, WFD VM placement with VM migration, FFD VM placement with VM migration, and BFD VM placement with VM migration, respectively).

In each of these 6 folders, you can find 8 different files. For example in the SpaceShare, TimeShare, and Hybrid folders you find these files: “Constants.java”, “GoogleClusterUtilizationModel.java”, “MyCloudlet.java”, “MyDatacenter.java”, “MyDatacenterBroker.java”, “MyVm.java”, “WfdVmAllocationPolicy.java”, and “Main.java”. The actual CloudSim code files are located in the “sources\org\cloudbus\cloudsim” path of the CloudSim package. Each of these 8 different files add something to one of these CloudSim code files, or modify some parts of them.

For example, the queuing theory part of our project is added to CloudSim PowerVm.java file. SO, open the MyVm.java file of our project (in the Hybrid, TimeShare, or SpaceShare folder open the MyVm.java file). You would see that it inherits from PowerVm.java file of CloudSim. Now look at the updateYmax function to find our code for updating $\lambda_{max}$ using the queuing theory method explained in the manuscript.

Or for example, M/M/m is used for hybrid and space-shared policies and M/M/1 is used for time-shared policies. Accordingly, If you open the “Constants.java” file of the Hybrid, SpaceShare, and TimeShare projects you would see that in the Hybrid and SpaceShare projects the VMs use m=2 cores (VM_PES) and in the TimeShare project the VMs use only 1 core (VM_PES).

As another example, the job scheduler part of our project is jointly implemented in the MyDatacenter.java and MyDatacenterBroker.java files of the Hybrid, SpaceShare, and TimeShare projects. Comments are added to the code, so you can follow each part and find out what is being done.

Now, we explain the inputs and outputs to our code. As explained in the manuscript, for the Hybrid, SpaceShare, and TimeShare projects the inputs are the maximum waiting time, and the probability bound; and the outputs are the total energy consumption and the normalized SLA violation. Moreover, for dynamicWfd, dynamicFfd, and dynamicBfd projects the input is the maximum waiting time and the outputs are the total energy consumption, the normalized SLA violation, and the number of migrations that took place. The main body of our projects can be found in “Main.java”. Open “Main.java” of one of the Hybrid, SpaceShare, or TimeShare projects. The first line in “public class Main” is:

// Replace this with your path to cloudsim folder.

public static String path = "C:/Users/Maryam/desktop/cloudsim-3.0.3";

So, change the value of path to the location of cloudsim-3.0.3 folder on your computer. Among the next variables there are:

public static double waitingTime = 0.1; //Sec.

public static double b = 0.8; // Probability bound

You can set the maximum waiting time and the probability bound of the project by modifying these two variables. So, that is how you can set the input variables. Additionally, after you execute the project (it would take 1 to 2 minutes for Hybrid, SpaceShare, and TimeShare projects to execute) the outputs are printed on the command prompt screen.

Similarly, for dynamicWfd, dynamicFfd, and dynamicBfd projects, the first line is:

// Replace this with your path to cloudsim folder.

public static String path = "C:/Users/Maryam/desktop/cloudsim-3.0.3";

You have to change the path variable to the location of CloudSim-3.0.3 folder on your computer. And then there is:

public static double waitingTime = 0.1; //sec

By changing waitingTime variable you can change the maximum waiting time of the project. It takes a little longer for dynamicWfd, dynamicFfd, and dynamicBfd to execute (several hours, maybe more than 12 hours). Finally, the outputs of these projects are also printed on the command prompt screen after the simulation completes.

We have used random variables in our code, therefore, every time you execute the simulation, the results might be slightly different. To accurately find the true results of our Hybrid, SpaceShare, and TimeShare projects, for each set of inputs we executed the simulation 10 times, then we calculated their average, and finally we drew the diagrams of our manuscript. In the tables below, you can find the 10 different values that we achieved for each experiment:

Since the dynamicWfd, dynamicFfd, and dynamicBfd projects took a very long time to execute, we simulated each scenario only 2 times, then we found their average, and eventually we drew the diagrams of our manuscript. Tables below show the results (b is the probability bound and W is the waiting time in seconds):

| Hybrid Policy  b=0.99 | | 1^st^ Trial | 2^nd^ Trial | 3^rd^ Trial | 4^th^ Trial | 5^th^ Trial | 6^th^ Trial | 7^th^ Trial | 8^th^ Trial | 9^th^ Trial | 10^th^ Trial | Average |
| --- | --- | --- | --- | --- | --- | --- | --- | --- | --- | --- | --- | --- |
| W=0.1 (Sec.) | Energy(KWh)  Normalized SLA | 0.8603  0.0 | 0.8603  0.0 | 0.8603  0.0 | 0.8603  0.0 | 0.8603  0.0 | 0.8603  0.0 | 0.8603  0.0 | 0.8603  0.0 | 0.8603  0.0 | 0.8603  0.0 | 0.8603  0.0 |
| W=0.2 (Sec.) | Energy(KWh)  Normalized SLA | 0.7801  0.0 | 0.7801  0.0 | 0.7801  0.0 | 0.7801  0.0 | 0.7801  0.0 | 0.7801  0.0 | 0.7801  0.0 | 0.7801  0.0 | 0.7801  0.0 | 0.7801  0.0 | 0.7801  0.0 |
| W=0.3 (Sec.) | Energy(KWh)  Normalized SLA | 0.7247  0.0 | 0.7247  0.0 | 0.7247  0.0 | 0.7247  0.0 | 0.7247  0.0 | 0.7247  0.0 | 0.7247  0.0 | 0.7247  0.0 | 0.7247  0.0 | 0.7247  0.0 | 0.7247  0.0 |
| W=0.4 (Sec.) | Energy(KWh)  Normalized SLA | 0.722  0.0 | 0.722  0.0 | 0.722  0.0 | 0.722  0.0 | 0.722  0.0 | 0.722  0.0 | 0.722  0.0 | 0.722  0.0 | 0.722  0.0 | 0.722  0.0 | 0.722  0.0 |
| W=0.5 (Sec.) | Energy(KWh)  Normalized SLA | 0.7164  0.0 | 0.7164  0.0 | 0.7164  0.0 | 0.7164  0.0 | 0.7164  0.0 | 0.7164  0.0 | 0.7164  0.0 | 0.7164  0.0 | 0.7164  0.0 | 0.7164  0.0 | 0.7164  0.0 |
| W=0.6 (Sec.) | Energy(KWh)  Normalized SLA | 0.7115  0.0 | 0.7115  0.0 | 0.7115  0.0 | 0.7115  0.0 | 0.7115  0.0 | 0.7115  0.0 | 0.7115  0.0 | 0.7115  0.0 | 0.7115  0.0 | 0.7115  0.0 | 0.7115  0.0 |
| W=0.7 (Sec.) | Energy(KWh)  Normalized SLA | 0.71  0.0 | 0.71  0.0 | 0.71  0.0 | 0.71  0.0 | 0.71  0.0 | 0.71  0.0 | 0.71  0.0 | 0.71  0.0 | 0.71  0.0 | 0.71  0.0 | 0.71  0.0 |
| W=0.8 (Sec.) | Energy(KWh)  Normalized SLA | 0.7208  0.0 | 0.7208  0.0 | 0.7208  0.0 | 0.7017  0.0 | 0.7112  0.0 | 0.7208  0.0 | 0.71  0.0 | 0.7208  0.0 | 0.7009  0.0 | 0.7208  0.0 | 0.71486  0.0 |
| W=0.9 (Sec.) | Energy(KWh)  Normalized SLA | 0.7087  0.0 | 0.7087  0.0 | 0.7087  0.0 | 0.7087  0.0 | 0.7087  0.0 | 0.7087  0.0 | 0.7087  0.0 | 0.7087  0.0 | 0.7087  0.0 | 0.7087  0.0 | 0.7087  0.0 |
| W=1 (Sec.) | Energy(KWh)  Normalized SLA | 0.7077  0.0 | 0.7077  0.0 | 0.7077  0.0 | 0.7077  0.0 | 0.7077  0.0 | 0.7077  0.0 | 0.7077  0.0 | 0.7077  0.0 | 0.7077  0.0 | 0.7077  0.0 | 0.7077  0.0 |

| Space-Shared Policy  b=0.99 | | 1^st^ Trial | 2^nd^ Trial | 3^rd^ Trial | 4^th^ Trial | 5^th^ Trial | 6^th^ Trial | 7^th^ Trial | 8^th^ Trial | 9^th^ Trial | 10^th^ Trial | Average |
| --- | --- | --- | --- | --- | --- | --- | --- | --- | --- | --- | --- | --- |
| W=0.1 (Sec.) | Energy(KWh)  Normalized SLA | 0.8989  0.0005 | 0.9013  0.002 | 0.8994  0.001 | 0.8623  0.0005 | 0.777  0.007 | 0.8589  0.0055 | 0.8605  0.0045 | 0.8992  0.001 | 0.8817  0.0035 | 0.8968  0.003 | 0.8736  0.00285 |
| W=0.2 (Sec.) | Energy(KWh)  Normalized SLA | 0.8955  0.0065 | 0.8478  0.0 | 0.8595  0.0005 | 0.8949  0.006 | 0.8981  0.0025 | 0.8972  0.002 | 0.8595  0.001 | 0.8679  0.0015 | 0.8596  0.0015 | 0.855  0.0065 | 0.8735  0.0028 |
| W=0.3 (Sec.) | Energy(KWh)  Normalized SLA | 0.842  0.0015 | 0.8423  0.001 | 0.8399  0.004 | 0.8457  0.0025 | 0.8395  0.003 | 0.8426  0.0015 | 0.8366  0.005 | 0.8427  0.0015 | 0.841  0.002 | 0.8497  0.0045 | 0.8422  0.00265 |
| W=0.4 (Sec.) | Energy(KWh)  Normalized SLA | 0.722  0.0025 | 0.7231  0.002 | 0.696  0.005 | 0.7225  0.0025 | 0.7252  0.0025 | 0.7191  0.003 | 0.7296  0.002 | 0.7331  0.002 | 0.7249  0.002 | 0.7269  0.002 | 0.72224  0.00255 |
| W=0.5 (Sec.) | Energy(KWh)  Normalized SLA | 0.723  0.0035 | 0.721  0.0035 | 0.7212  0.002 | 0.7202  0.002 | 0.729  0.0025 | 0.7182  0.002 | 0.7217  0.002 | 0.7217  0.0025 | 0.7217  0.0025 | 0.7217  0.002 | 0.72194  0.00245 |
| W=0.6 (Sec.) | Energy(KWh)  Normalized SLA | 0.7213  0.0025 | 0.7212  0.0025 | 0.7208  0.002 | 0.725  0.002 | 0.6892  0.003 | 0.721  0.002 | 0.7194  0.0035 | 0.7212  0.002 | 0.7212  0.002 | 0.7212  0.0025 | 0.71815  0.0024 |
| W=0.7 (Sec.) | Energy(KWh)  Normalized SLA | 0.7239  0.003 | 0.7239  0.0025 | 0.7199  0.003 | 0.7199  0.0025 | 0.719  0.0025 | 0.6794  0.003 | 0.7247  0.0025 | 0.7183  0.0025 | 0.7199  0.0025 | 0.7187  0.001 | 0.71676  0.0025 |
| W=0.8 (Sec.) | Energy(KWh)  Normalized SLA | 0.7182  0.0015 | 0.7225  0.002 | 0.7177  0.002 | 0.7182  0.0015 | 0.717  0.0025 | 0.6855  0.003 | 0.7176  0.002 | 0.717  0.0025 | 0.7168  0.003 | 0.7187  0.002 | 0.71492  0.0022 |
| W=0.9 (Sec.) | Energy(KWh)  Normalized SLA | 0.7146  0.0025 | 0.7147  0.0015 | 0.7163  0.0025 | 0.7156  0.001 | 0.6763  0.0015 | 0.7136  0.003 | 0.7147  0.0015 | 0.7146  0.0025 | 0.7147  0.002 | 0.7147  0.002 | 0.71098  0.0022 |
| W=1 (Sec.) | Energy(KWh)  Normalized SLA | 0.7134  0.003 | 0.7093  0.003 | 0.7094  0.002 | 0.7134  0.0025 | 0.7094  0.001 | 0.7187  0.002 | 0.7134  0.0025 | 0.7115  0.002 | 0.7187  0.002 | 0.712  0.0025 | 0.71297  0.00225 |

| Time-Shared Policy  b=0.99 | | 1^st^ Trial | 2^nd^ Trial | 3^rd^ Trial | 4^th^ Trial | 5^th^ Trial | 6^th^ Trial | 7^th^ Trial | 8^th^ Trial | 9^th^ Trial | 10^th^ Trial | Average |
| --- | --- | --- | --- | --- | --- | --- | --- | --- | --- | --- | --- | --- |
| W=0.1 (Sec.) | Energy(KWh)  Normalized SLA | 1.0602  0.0 | 1.0602  0.0 | 1.0602  0.0 | 1.0602  0.0 | 1.0602  0.0 | 1.0602  0.0 | 1.0602  0.0 | 1.0602  0.0 | 1.0602  0.0 | 1.0602  0.0 | 1.0602  0.0 |
| W=0.2 (Sec.) | Energy(KWh)  Normalized SLA | 1.0602  0.0 | 1.0602  0.0 | 1.0602  0.0 | 1.0602  0.0 | 1.0602  0.0 | 1.0602  0.0 | 1.0602  0.0 | 1.0602  0.0 | 1.0602  0.0 | 1.0602  0.0 | 1.0602  0.0 |
| W=0.3 (Sec.) | Energy(KWh)  Normalized SLA | 1.0602  0.0 | 1.0602  0.0 | 1.0602  0.0 | 1.0602  0.0 | 1.0602  0.0 | 1.0602  0.0 | 1.0602  0.0 | 1.0602  0.0 | 1.0602  0.0 | 1.0602  0.0 | 1.0602  0.0 |
| W=0.4 (Sec.) | Energy(KWh)  Normalized SLA | 1.0602  0.0 | 1.0602  0.0 | 1.0602  0.0 | 1.0602  0.0 | 1.0602  0.0 | 1.0602  0.0 | 1.0602  0.0 | 1.0602  0.0 | 1.0602  0.0 | 1.0602  0.0 | 1.0602  0.0 |
| W=0.5 (Sec.) | Energy(KWh)  Normalized SLA | 1.0602  0.0 | 1.0602  0.0 | 1.0602  0.0 | 1.0602  0.0 | 1.0602  0.0 | 1.0602  0.0 | 1.0602  0.0 | 1.0602  0.0 | 1.0602  0.0 | 1.0602  0.0 | 1.0602  0.0 |
| W=0.6 (Sec.) | Energy(KWh)  Normalized SLA | 1.0602  0.0 | 1.0602  0.0 | 1.0602  0.0 | 1.0602  0.0 | 1.0602  0.0 | 1.0602  0.0 | 1.0602  0.0 | 1.0602  0.0 | 1.0602  0.0 | 1.0602  0.0 | 1.0602  0.0 |
| W=0.7 (Sec.) | Energy(KWh)  Normalized SLA | 1.0602  0.0 | 1.0602  0.0 | 1.0602  0.0 | 1.0602  0.0 | 1.0602  0.0 | 1.0602  0.0 | 1.0602  0.0 | 1.0602  0.0 | 1.0602  0.0 | 1.0602  0.0 | 1.0602  0.0 |
| W=0.8 (Sec.) | Energy(KWh)  Normalized SLA | 1.0602  0.0 | 1.0602  0.0 | 1.0602  0.0 | 1.0602  0.0 | 1.0602  0.0 | 1.0602  0.0 | 1.0602  0.0 | 1.0602  0.0 | 1.0602  0.0 | 1.0602  0.0 | 1.0602  0.0 |
| W=0.9 (Sec.) | Energy(KWh)  Normalized SLA | 1.0602  0.0 | 1.0602  0.0 | 1.0602  0.0 | 1.0602  0.0 | 1.0602  0.0 | 1.0602  0.0 | 1.0602  0.0 | 1.0602  0.0 | 1.0602  0.0 | 1.0602  0.0 | 1.0602  0.0 |
| W=1 (Sec.) | Energy(KWh)  Normalized SLA | 1.0602  0.0 | 1.0602  0.0 | 1.0602  0.0 | 1.0602  0.0 | 1.0602  0.0 | 1.0602  0.0 | 1.0602  0.0 | 1.0602  0.0 | 1.0602  0.0 | 1.0602  0.0 | 1.0602  0.0 |

| Hybrid Policy  b=0.9 | | 1^st^ Trial | 2^nd^ Trial | 3^rd^ Trial | 4^th^ Trial | 5^th^ Trial | 6^th^ Trial | 7^th^ Trial | 8^th^ Trial | 9^th^ Trial | 10^th^ Trial | Average |
| --- | --- | --- | --- | --- | --- | --- | --- | --- | --- | --- | --- | --- |
| W=0.1 (Sec.) | Energy(KWh)  Normalized SLA | 0.8603  0.0 | 0.8603  0.0 | 0.8603  0.0 | 0.8603  0.0 | 0.8603  0.0 | 0.8603  0.0 | 0.8603  0.0 | 0.8603  0.0 | 0.8603  0.0 | 0.8603  0.0 | 0.8603  0.0 |
| W=0.2 (Sec.) | Energy(KWh)  Normalized SLA | 0.7801  0.0 | 0.7801  0.0 | 0.7801  0.0 | 0.7801  0.0 | 0.7801  0.0 | 0.7801  0.0 | 0.7801  0.0 | 0.7801  0.0 | 0.7801  0.0 | 0.7801  0.0 | 0.7801  0.0 |
| W=0.3 (Sec.) | Energy(KWh)  Normalized SLA | 0.7247  0.0 | 0.7247  0.0 | 0.7247  0.0 | 0.7247  0.0 | 0.7247  0.0 | 0.7247  0.0 | 0.7247  0.0 | 0.7247  0.0 | 0.7247  0.0 | 0.7247  0.0 | 0.7247  0.0 |
| W=0.4 (Sec.) | Energy(KWh)  Normalized SLA | 0.722  0.0 | 0.722  0.0 | 0.722  0.0 | 0.722  0.0 | 0.722  0.0 | 0.722  0.0 | 0.722  0.0 | 0.722  0.0 | 0.722  0.0 | 0.722  0.0 | 0.722  0.0 |
| W=0.5 (Sec.) | Energy(KWh)  Normalized SLA | 0.71  0.0 | 0.71  0.0 | 0.71  0.0 | 0.71  0.0 | 0.71  0.0 | 0.71  0.0 | 0.71  0.0 | 0.71  0.0 | 0.71  0.0 | 0.71  0.0 | 0.71  0.0 |
| W=0.6 (Sec.) | Energy(KWh)  Normalized SLA | 0.7115  0.0 | 0.7115  0.0 | 0.7115  0.0 | 0.7115  0.0 | 0.7115  0.0 | 0.7115  0.0 | 0.7115  0.0 | 0.7115  0.0 | 0.7115  0.0 | 0.7115  0.0 | 0.7115  0.0 |
| W=0.7 (Sec.) | Energy(KWh)  Normalized SLA | 0.71  0.0 | 0.71  0.0 | 0.71  0.0 | 0.71  0.0 | 0.71  0.0 | 0.71  0.0 | 0.71  0.0 | 0.71  0.0 | 0.71  0.0 | 0.71  0.0 | 0.71  0.0 |
| W=0.8 (Sec.) | Energy(KWh)  Normalized SLA | 0.7208  0.00 | 0.7208  0.00 | 0.7189  0.00 | 0.7026  0.00 | 0.7208  0.00 | 0.7018  0.00 | 0.7208  0.00 | 0.7208  0.00 | 0.7208  0.00 | 0.7139  0.00 | 0.7162  0.00 |
| W=0.9 (Sec.) | Energy(KWh)  Normalized SLA | 0.7087  0.0 | 0.7087  0.0 | 0.7087  0.0 | 0.7087  0.0 | 0.7087  0.0 | 0.7087  0.0 | 0.7087  0.0 | 0.7087  0.0 | 0.7087  0.0 | 0.7087  0.0 | 0.7087  0.0 |
| W=1 (Sec.) | Energy(KWh)  Normalized SLA | 0.7077  0.0 | 0.7077  0.0 | 0.7077  0.0 | 0.7077  0.0 | 0.7077  0.0 | 0.7077  0.0 | 0.7077  0.0 | 0.7077  0.0 | 0.7077  0.0 | 0.7077  0.0 | 0.7077  0.0 |

| Space-Shared Policy  b=0.9 | | 1^st^ Trial | 2^nd^ Trial | 3^rd^ Trial | 4^th^ Trial | 5^th^ Trial | 6^th^ Trial | 7^th^ Trial | 8^th^ Trial | 9^th^ Trial | 10^th^ Trial | Average |
| --- | --- | --- | --- | --- | --- | --- | --- | --- | --- | --- | --- | --- |
| W=0.1 (Sec.) | Energy(KWh)  Normalized SLA | 0.8997  0.0 | 0.8997  0.0 | 0.8997  0.0 | 0.8997  0.0 | 0.8997  0.0 | 0.8997  0.0 | 0.8997  0.0 | 0.8997  0.0 | 0.8997  0.0 | 0.8997  0.0 | 0.8997  0.0 |
| W=0.2 (Sec.) | Energy(KWh)  Normalized SLA | 0.8833  0.0005 | 0.8833  0.0005 | 0.8833  0.0005 | 0.8833  0.0005 | 0.8833  0.0005 | 0.8833  0.0005 | 0.8833  0.0005 | 0.8833  0.0005 | 0.8833  0.0005 | 0.8833  0.0005 | 0.8833  0.0005 |
| W=0.3 (Sec.) | Energy(KWh)  Normalized SLA | 0.7249  0.0015 | 0.7247  0.0025 | 0.7249  0.0015 | 0.7249  0.0015 | 0.7249  0.0015 | 0.7249  0.0015 | 0.7248  0.0015 | 0.7255  0.0015 | 0.7249  0.0015 | 0.7249  0.0015 | 0.72493  0.0016 |
| W=0.4 (Sec.) | Energy(KWh)  Normalized SLA | 0.7248  0.002 | 0.7248  0.002 | 0.7248  0.0015 | 0.7248  0.001 | 0.7203  0.002 | 0.7268  0.001 | 0.7248  0.001 | 0.7248  0.001 | 0.7248  0.001 | 0.7234  0.002 | 0.72441  0.00145 |
| W=0.5 (Sec.) | Energy(KWh)  Normalized SLA | 0.7218  0.0025 | 0.7204  0.0025 | 0.7204  0.0015 | 0.6926  0.001 | 0.7223  0.002 | 0.7223  0.001 | 0.7223  0.001 | 0.7223  0.002 | 0.7223  0.001 | 0.7223  0.0015 | 0.7189  0.0016 |
| W=0.6 (Sec.) | Energy(KWh)  Normalized SLA | 0.7197  0.002 | 0.7189  0.002 | 0.7189  0.001 | 0.7189  0.001 | 0.7189  0.001 | 0.7197  0.001 | 0.7189  0.001 | 0.7189  0.002 | 0.7189  0.001 | 0.7189  0.001 | 0.71906  0.0013 |
| W=0.7 (Sec.) | Energy(KWh)  Normalized SLA | 0.7185  0.002 | 0.6806  0.002 | 0.7181  0.0025 | 0.7185  0.001 | 0.7224  0.0005 | 0.7185  0.001 | 0.7185  0.001 | 0.7185  0.001 | 0.7181  0.0015 | 0.7185  0.001 | 0.71502  0.00135 |
| W=0.8 (Sec.) | Energy(KWh)  Normalized SLA | 0.7148  0.002 | 0.7148  0.002 | 0.7148  0.0015 | 0.7242  0.0025 | 0.7242  0.0015 | 0.7148  0.001 | 0.6892  0.001 | 0.7306  0.0005 | 0.7148  0.001 | 0.721  0.001 | 0.71632  0.0014 |
| W=0.9 (Sec.) | Energy(KWh)  Normalized SLA | 0.7128  0.003 | 0.7129  0.0025 | 0.7129  0.0015 | 0.7129  0.001 | 0.7128  0.002 | 0.7129  0.001 | 0.7129  0.001 | 0.7128  0.002 | 0.7129  0.001 | 0.7129  0.001 | 0.71288  0.0016 |
| W=1 (Sec.) | Energy(KWh)  Normalized SLA | 0.7117  0.0035 | 0.7129  0.0025 | 0.7129  0.001 | 0.7129  0.002 | 0.7129  0.0025 | 0.7129  0.001 | 0.7129  0.001 | 0.7129  0.001 | 0.7129  0.002 | 0.7129  0.001 | 0.71278  0.00175 |

| Time-Shared Policy  b=0.9 | | 1^st^ Trial | 2^nd^ Trial | 3^rd^ Trial | 4^th^ Trial | 5^th^ Trial | 6^th^ Trial | 7^th^ Trial | 8^th^ Trial | 9^th^ Trial | 10^th^ Trial | Average |
| --- | --- | --- | --- | --- | --- | --- | --- | --- | --- | --- | --- | --- |
| W=0.1 (Sec.) | Energy(KWh)  Normalized SLA | 1.0602  0.0 | 1.0602  0.0 | 1.0602  0.0 | 1.0602  0.0 | 1.0602  0.0 | 1.0602  0.0 | 1.0602  0.0 | 1.0602  0.0 | 1.0602  0.0 | 1.0602  0.0 | 1.0602  0.0 |
| W=0.2 (Sec.) | Energy(KWh)  Normalized SLA | 1.0602  0.0 | 1.0602  0.0 | 1.0602  0.0 | 1.0602  0.0 | 1.0602  0.0 | 1.0602  0.0 | 1.0602  0.0 | 1.0602  0.0 | 1.0602  0.0 | 1.0602  0.0 | 1.0602  0.0 |
| W=0.3 (Sec.) | Energy(KWh)  Normalized SLA | 1.0973  0.0 | 1.0973  0.0 | 1.0973  0.0 | 1.0973  0.0 | 1.0973  0.0 | 1.0973  0.0 | 1.0973  0.0 | 1.0973  0.0 | 1.0973  0.0 | 1.0973  0.0 | 1.0973  0.0 |
| W=0.4 (Sec.) | Energy(KWh)  Normalized SLA | 1.0973  0.0 | 1.0973  0.0 | 1.0973  0.0 | 1.0973  0.0 | 1.0973  0.0 | 1.0973  0.0 | 1.0973  0.0 | 1.0973  0.0 | 1.0973  0.0 | 1.0973  0.0 | 1.0973  0.0 |
| W=0.5 (Sec.) | Energy(KWh)  Normalized SLA | 1.105  0.0 | 1.105  0.0 | 1.105  0.0 | 1.105  0.0 | 1.105  0.0 | 1.105  0.0 | 1.105  0.0 | 1.105  0.0 | 1.105  0.0 | 1.105  0.0 | 1.105  0.0 |
| W=0.6 (Sec.) | Energy(KWh)  Normalized SLA | 1.0603  0.0 | 1.0603  0.0 | 1.0603  0.0 | 1.0603  0.0 | 1.0603  0.0 | 1.0603  0.0 | 1.0603  0.0 | 1.0603  0.0 | 1.0603  0.0 | 1.0603  0.0 | 1.0603  0.0 |
| W=0.7 (Sec.) | Energy(KWh)  Normalized SLA | 1.0459  0.0 | 1.0459  0.0 | 1.0459  0.0 | 1.0459  0.0 | 1.0459  0.0 | 1.0459  0.0 | 1.0459  0.0 | 1.0459  0.0 | 1.0459  0.0 | 1.0459  0.0 | 1.0459  0.0 |
| W=0.8 (Sec.) | Energy(KWh)  Normalized SLA | 0.9448  0.0 | 0.948  0.0 | 0.9448  0.0 | 0.948  0.0 | 0.9448  0.0 | 0.948  0.0 | 0.9448  0.0 | 0.948  0.0 | 0.9448  0.0 | 0.9448  0.0 | 0.948  0.0 |
| W=0.9 (Sec.) | Energy(KWh)  Normalized SLA | 0.9458  0.0 | 0.9458  0.0 | 0.9458  0.0 | 0.9458  0.0 | 0.9458  0.0 | 0.9458  0.0 | 0.9458  0.0 | 0.9458  0.0 | 0.9458  0.0 | 0.9458  0.0 | 0.9458  0.0 |
| W=1 (Sec.) | Energy(KWh)  Normalized SLA | 0.9422  0.0 | 0.9422  0.0 | 0.9422  0.0 | 0.9422  0.0 | 0.9422  0.0 | 0.9422  0.0 | 0.9422  0.0 | 0.9422  0.0 | 0.9422  0.0 | 0.9422  0.0 | 0.9422  0.0 |

| Hybrid Policy  b=0.8 | | 1^st^ Trial | 2^nd^ Trial | 3^rd^ Trial | 4^th^ Trial | 5^th^ Trial | 6^th^ Trial | 7^th^ Trial | 8^th^ Trial | 9^th^ Trial | 10^th^ Trial | Average |
| --- | --- | --- | --- | --- | --- | --- | --- | --- | --- | --- | --- | --- |
| W=0.1 (Sec.) | Energy(KWh)  Normalized SLA | 0.8976  0.0 | 0.8976  0.0 | 0.8967  0.0 | 0.8976  0.0 | 0.8976  0.0 | 0.8967  0.0 | 0.8976  0.0 | 0.8976  0.0 | 0.8976  0.0 | 0.8967  0.0 | 0.89742  0.0 |
| W=0.2 (Sec.) | Energy(KWh)  Normalized SLA | 0.7639  0.0 | 0.7639  0.0 | 0.7639  0.0 | 0.7639  0.0 | 0.7639  0.0 | 0.7639  0.0 | 0.7639  0.0 | 0.7639  0.0 | 0.7639  0.0 | 0.7639  0.0 | 0.7639  0.0 |
| W=0.3 (Sec.) | Energy(KWh)  Normalized SLA | 0.7903  0.0 | 0.7903  0.0 | 0.7621  0.0 | 0.7266  0.0 | 0.7903  0.0 | 0.7266  0.0 | 0.7012  0.0 | 0.7903  0.0 | 0.7266  0.0 | 0.701  0.0 | 0.75054  0.0 |
| W=0.4 (Sec.) | Energy(KWh)  Normalized SLA | 0.722  0.0 | 0.722  0.0 | 0.722  0.0 | 0.722  0.0 | 0.722  0.0 | 0.722  0.0 | 0.722  0.0 | 0.722  0.0 | 0.722  0.0 | 0.722  0.0 | 0.722  0.0 |
| W=0.5 (Sec.) | Energy(KWh)  Normalized SLA | 0.71  0.0 | 0.71  0.0 | 0.71  0.0 | 0.71  0.0 | 0.71  0.0 | 0.71  0.0 | 0.71  0.0 | 0.71  0.0 | 0.71  0.0 | 0.71  0.0 | 0.71  0.0 |
| W=0.6 (Sec.) | Energy(KWh)  Normalized SLA | 0.7115  0.0 | 0.7115  0.0 | 0.7115  0.0 | 0.7115  0.0 | 0.7115  0.0 | 0.7115  0.0 | 0.7115  0.0 | 0.7115  0.0 | 0.7115  0.0 | 0.7115  0.0 | 0.7115  0.0 |
| W=0.7 (Sec.) | Energy(KWh)  Normalized SLA | 0.71  0.0 | 0.71  0.0 | 0.71  0.0 | 0.71  0.0 | 0.71  0.0 | 0.71  0.0 | 0.71  0.0 | 0.71  0.0 | 0.71  0.0 | 0.71  0.0 | 0.71  0.0 |
| W=0.8 (Sec.) | Energy(KWh)  Normalized SLA | 0.7208  0.0 | 0.7208  0.0 | 0.7208  0.0 | 0.7208  0.0 | 0.7208  0.0 | 0.7208  0.0 | 0.7208  0.0 | 0.7208  0.0 | 0.7208  0.0 | 0.7208  0.0 | 0.7208  0.0 |
| W=0.9 (Sec.) | Energy(KWh)  Normalized SLA | 0.7087  0.0 | 0.7087  0.0 | 0.7087  0.0 | 0.7087  0.0 | 0.7087  0.0 | 0.7087  0.0 | 0.7087  0.0 | 0.7087  0.0 | 0.7087  0.0 | 0.7087  0.0 | 0.7087  0.0 |
| W=1 (Sec.) | Energy(KWh)  Normalized SLA | 0.7077  0.0 | 0.7077  0.0 | 0.7077  0.0 | 0.7077  0.0 | 0.7077  0.0 | 0.7077  0.0 | 0.7077  0.0 | 0.7077  0.0 | 0.7077  0.0 | 0.7077  0.0 | 0.7077  0.0 |

| Space-Shared Policy  b=0.8 | | 1^st^ Trial | 2^nd^ Trial | 3^rd^ Trial | 4^th^ Trial | 5^th^ Trial | 6^th^ Trial | 7^th^ Trial | 8^th^ Trial | 9^th^ Trial | 10^th^ Trial | Average |
| --- | --- | --- | --- | --- | --- | --- | --- | --- | --- | --- | --- | --- |
| W=0.1 (Sec.) | Energy(KWh)  Normalized SLA | 0.8997  0.0 | 0.8997  0.0 | 0.8997  0.0 | 0.8997  0.0 | 0.8997  0.0 | 0.8997  0.0 | 0.8997  0.0 | 0.8997  0.0 | 0.8997  0.0 | 0.8997  0.0 | 0.8997  0.0 |
| W=0.2 (Sec.) | Energy(KWh)  Normalized SLA | 0.8463  0.0005 | 0.8463  0.0005 | 0.8463  0.0005 | 0.8463  0.0005 | 0.8463  0.0005 | 0.8463  0.0005 | 0.8463  0.0005 | 0.8463  0.0005 | 0.8463  0.0005 | 0.8463  0.0005 | 0.8463  0.0005 |
| W=0.3 (Sec.) | Energy(KWh)  Normalized SLA | 0.7251  0.0025 | 0.7251  0.0025 | 0.7251  0.0015 | 0.7867  0.002 | 0.7701  0.0015 | 0.7251  0.0015 | 0.7701  0.0015 | 0.7867  0.002 | 0.7664  0.0015 | 0.7251  0.0015 | 0.75055  0.00175 |
| W=0.4 (Sec.) | Energy(KWh)  Normalized SLA | 0.7248  0.002 | 0.7248  0.002 | 0.7248  0.002 | 0.7248  0.001 | 0.7248  0.001 | 0.7248  0.002 | 0.7248  0.001 | 0.7248  0.001 | 0.7248  0.002 | 0.7248  0.001 | 0.7248  0.0015 |
| W=0.5 (Sec.) | Energy(KWh)  Normalized SLA | 0.7205  0.0025 | 0.7205  0.0025 | 0.7205  0.0015 | 0.7205  0.0025 | 0.7205  0.001 | 0.7205  0.0015 | 0.7205  0.0025 | 0.7205  0.0025 | 0.7205  0.0015 | 0.7205  0.0015 | 0.7205  0.00195 |
| W=0.6 (Sec.) | Energy(KWh)  Normalized SLA | 0.7201  0.0015 | 0.7201  0.0015 | 0.7201  0.0015 | 0.7201  0.0015 | 0.7201  0.0015 | 0.7201  0.0015 | 0.7201  0.0015 | 0.7201  0.0015 | 0.7201  0.0015 | 0.7201  0.0015 | 0.7201  0.0015 |
| W=0.7 (Sec.) | Energy(KWh)  Normalized SLA | 0.7173  0.002 | 0.7173  0.002 | 0.7173  0.001 | 0.7173  0.002 | 0.7173  0.001 | 0.7173  0.001 | 0.7173  0.002 | 0.7173  0.001 | 0.7173  0.002 | 0.7173  0.001 | 0.7173  0.0015 |
| W=0.8 (Sec.) | Energy(KWh)  Normalized SLA | 0.7168  0.0025 | 0.7168  0.0025 | 0.722  0.0015 | 0.7201  0.0015 | 0.7392  0.0015 | 0.7168  0.0025 | 0.7168  0.0015 | 0.7234  0.001 | 0.7201  0.0015 | 0.7168  0.0005 | 0.72088  0.00165 |
| W=0.9 (Sec.) | Energy(KWh)  Normalized SLA | 0.7133  0.0035 | 0.7134  0.0035 | 0.7134  0.0015 | 0.7134  0.001 | 0.7134  0.0015 | 0.7134  0.0035 | 0.7134  0.0015 | 0.7134  0.0035 | 0.7134  0.001 | 0.7134  0.0015 | 0.7134  0.0022 |
| W=1 (Sec.) | Energy(KWh)  Normalized SLA | 0.7107  0.004 | 0.7107  0.004 | 0.7107  0.002 | 0.7107  0.002 | 0.7107  0.004 | 0.7107  0.002 | 0.7107  0.002 | 0.7107  0.002 | 0.7107  0.004 | 0.7107  0.002 | 0.7107  0.0028 |

| Time-Shared Policy  b=0.8 | | 1^st^ Trial | 2^nd^ Trial | 3^rd^ Trial | 4^th^ Trial | 5^th^ Trial | 6^th^ Trial | 7^th^ Trial | 8^th^ Trial | 9^th^ Trial | 10^th^ Trial | Average |
| --- | --- | --- | --- | --- | --- | --- | --- | --- | --- | --- | --- | --- |
| W=0.1 (Sec.) | Energy(KWh)  Normalized SLA | 1.0602  0.0 | 1.0602  0.0 | 1.0602  0.0 | 1.0602  0.0 | 1.0602  0.0 | 1.0602  0.0 | 1.0602  0.0 | 1.0602  0.0 | 1.0602  0.0 | 1.0602  0.0 | 1.0602  0.0 |
| W=0.2 (Sec.) | Energy(KWh)  Normalized SLA | 1.0973  0.0 | 1.0973  0.0 | 1.0973  0.0 | 1.0973  0.0 | 1.0973  0.0 | 1.0973  0.0 | 1.0973  0.0 | 1.0973  0.0 | 1.0973  0.0 | 1.0973  0.0 | 1.0973  0.0 |
| W=0.3 (Sec.) | Energy(KWh)  Normalized SLA | 1.0614  0.0 | 1.0614  0.0 | 1.0614  0.0 | 1.0614  0.0 | 1.0614  0.0 | 1.0614  0.0 | 1.0614  0.0 | 1.0614  0.0 | 1.0614  0.0 | 1.0614  0.0 | 1.0614  0.0 |
| W=0.4 (Sec.) | Energy(KWh)  Normalized SLA | 1.0183  0.0 | 1.0183  0.0 | 1.0183  0.0 | 1.0183  0.0 | 1.0183  0.0 | 1.0183  0.0 | 1.0183  0.0 | 1.0183  0.0 | 1.0183  0.0 | 1.0183  0.0 | 1.0183  0.0 |
| W=0.5 (Sec.) | Energy(KWh)  Normalized SLA | 1.0216  0.001 | 1.0216  0.001 | 1.0216  0.001 | 1.0216  0.001 | 1.0216  0.001 | 1.0216  0.001 | 1.0216  0.001 | 1.0216  0.001 | 1.0216  0.001 | 1.0216  0.001 | 1.0216  0.001 |
| W=0.6 (Sec.) | Energy(KWh)  Normalized SLA | 0.9303  0.0 | 0.9303  0.0 | 0.9303  0.0 | 0.9303  0.0 | 0.9303  0.0 | 0.9303  0.0 | 0.9303  0.0 | 0.9303  0.0 | 0.9303  0.0 | 0.9303  0.0 | 0.9303  0.0 |
| W=0.7 (Sec.) | Energy(KWh)  Normalized SLA | 0.9494  0.0 | 0.9494  0.0 | 0.9494  0.0 | 0.9494  0.0 | 0.9494  0.0 | 0.9494  0.0 | 0.9494  0.0 | 0.9494  0.0 | 0.9494  0.0 | 0.9494  0.0 | 0.9494  0.0 |
| W=0.8 (Sec.) | Energy(KWh)  Normalized SLA | 0.9536  0.001 | 0.9536  0.001 | 0.9536  0.001 | 0.9536  0.001 | 0.9536  0.001 | 0.9536  0.001 | 0.9536  0.001 | 0.9536  0.001 | 0.9536  0.001 | 0.9536  0.001 | 0.9536  0.001 |
| W=0.9 (Sec.) | Energy(KWh)  Normalized SLA | 0.926  0.0 | 0.926  0.0 | 0.926  0.0 | 0.926  0.0 | 0.926  0.0 | 0.926  0.0 | 0.926  0.0 | 0.926  0.0 | 0.926  0.0 | 0.926  0.0 | 0.926  0.0 |
| W=1 (Sec.) | Energy(KWh)  Normalized SLA | 0.9227  0.001 | 0.9227  0.001 | 0.9227  0.001 | 0.9227  0.001 | 0.9227  0.001 | 0.9227  0.001 | 0.9227  0.001 | 0.9227  0.001 | 0.9227  0.001 | 0.9227  0.001 | 0.9227  0.001 |

Since dynamicBfd, dynamicFfd, and dynamicWfd took a very long time to run, we executed them only once for different waiting times:

| dynamicBfd | W=0.1 (Sec.) | W=0.2 (Sec.) | W=0.3 (Sec.) | W=0.4 (Sec.) | W=0.5 (Sec.) | W=0.6 (Sec.) | W=0.7 (Sec.) | W=0.8 (Sec.) | W=0.9 (Sec.) | W=1  (Sec.) |
| --- | --- | --- | --- | --- | --- | --- | --- | --- | --- | --- |
| Energy(KWh) | 1.3813 | 1.3813 | 1.3813 | 1.3813 | 1.3813 | 1.3813 | 1.3813 | 1.3813 | 1.3813 | 1.3813 |
| Normalized SLA | 0.009 | 0.009 | 0.0085 | 0.008 | 0.0075 | 0.0065 | 0.0055 | 0.005 | 0.0045 | 0.0045 |
| Number of Migrations | 167993 | 167993 | 167993 | 167993 | 167993 | 167993 | 167993 | 167993 | 167993 | 167993 |

| dynamicFfd | W=0.1 (Sec.) | W=0.2 (Sec.) | W=0.3 (Sec.) | W=0.4 (Sec.) | W=0.5 (Sec.) | W=0.6 (Sec.) | W=0.7 (Sec.) | W=0.8 (Sec.) | W=0.9 (Sec.) | W=1  (Sec.) |
| --- | --- | --- | --- | --- | --- | --- | --- | --- | --- | --- |
| Energy(KWh) | 0.9286 | 0.9286 | 0.9286 | 0.9286 | 0.9286 | 0.9286 | 0.9286 | 0.9286 | 0.9286 | 0.9286 |
| Normalized SLA | 0.011 | 0.011 | 0.011 | 0.0105 | 0.0105 | 0.01 | 0.009 | 0.008 | 0.007 | 0.006 |
| Number of Migrations | 327045 | 327045 | 327045 | 327045 | 327045 | 327045 | 327045 | 327045 | 327045 | 327045 |

| dynamicWfd | W=0.1 (Sec.) | W=0.2 (Sec.) | W=0.3 (Sec.) | W=0.4 (Sec.) | W=0.5 (Sec.) | W=0.6 (Sec.) | W=0.7 (Sec.) | W=0.8 (Sec.) | W=0.9 (Sec.) | W=1  (Sec.) |
| --- | --- | --- | --- | --- | --- | --- | --- | --- | --- | --- |
| Energy(KWh) | 0.9214 | 0.9214 | 0.9214 | 0.9214 | 0.9214 | 0.9214 | 0.9214 | 0.9214 | 0.9214 | 0.9214 |
| Normalized SLA | 0.014 | 0.014 | 0.014 | 0.0135 | 0.0135 | 0.0125 | 0.0115 | 0.01 | 0.009 | 0.008 |
| Number of Migrations | 344845 | 344845 | 344845 | 344845 | 344834 | 344845 | 344834 | 344845 | 344845 | 344834 |

According to the tables above, we find the diagrams below.


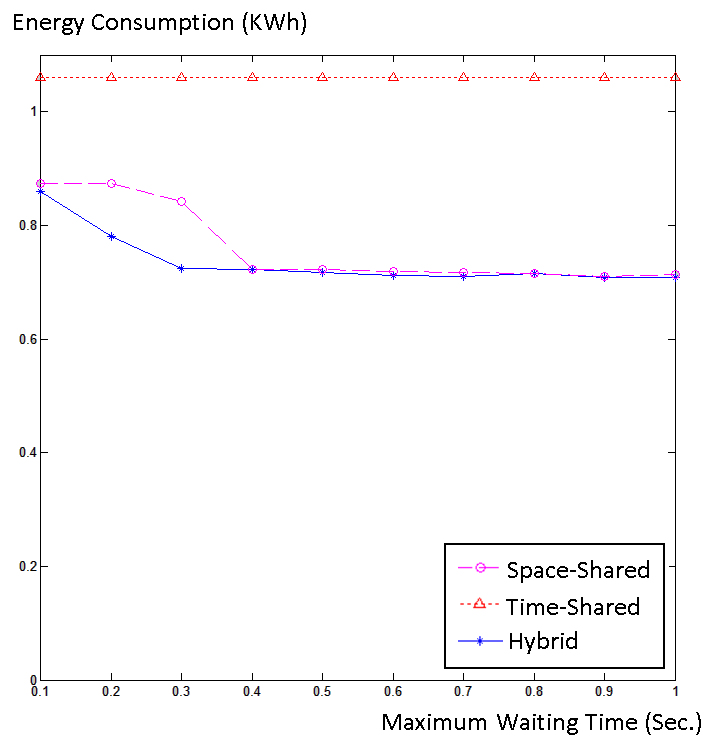

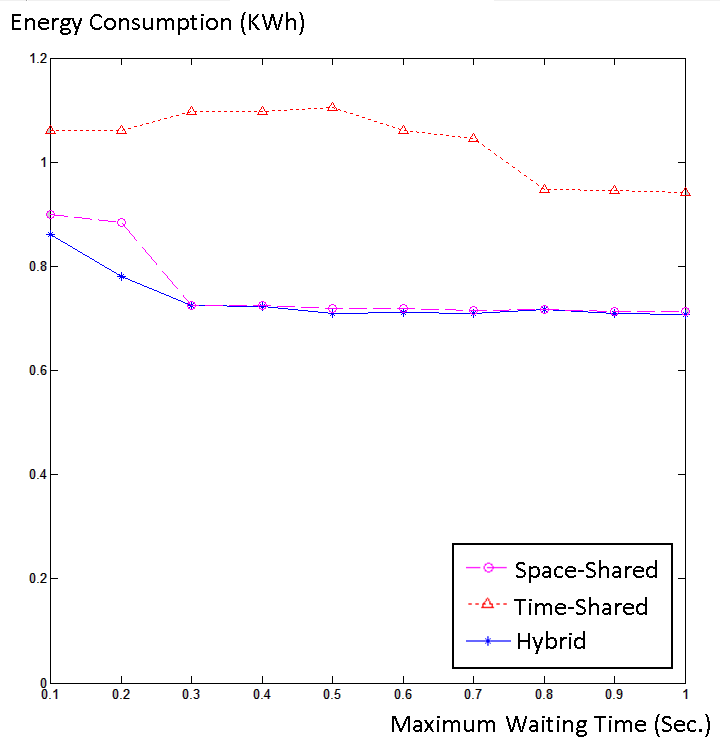

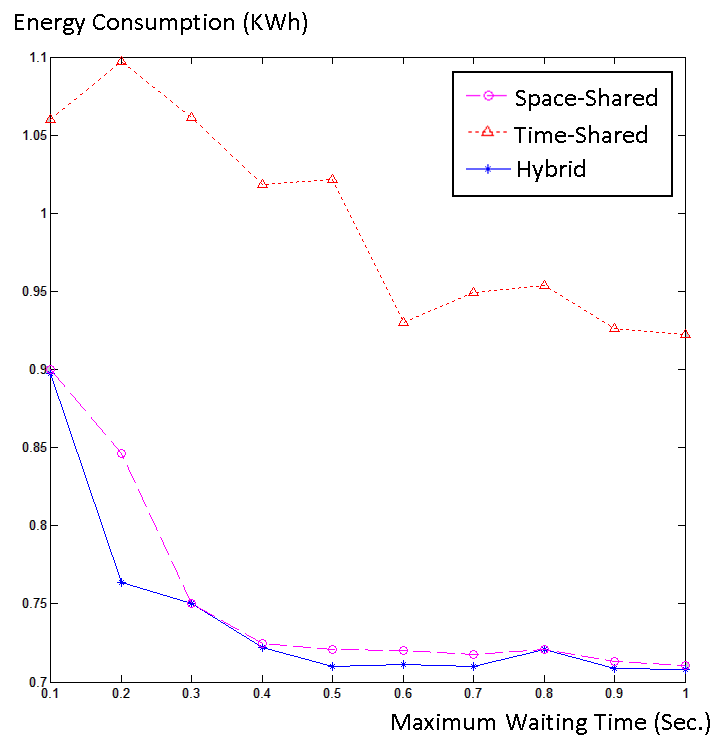


Figure 2a: b=0.99 Figure 2b: b=0.9 Figure 2c: b=0.8


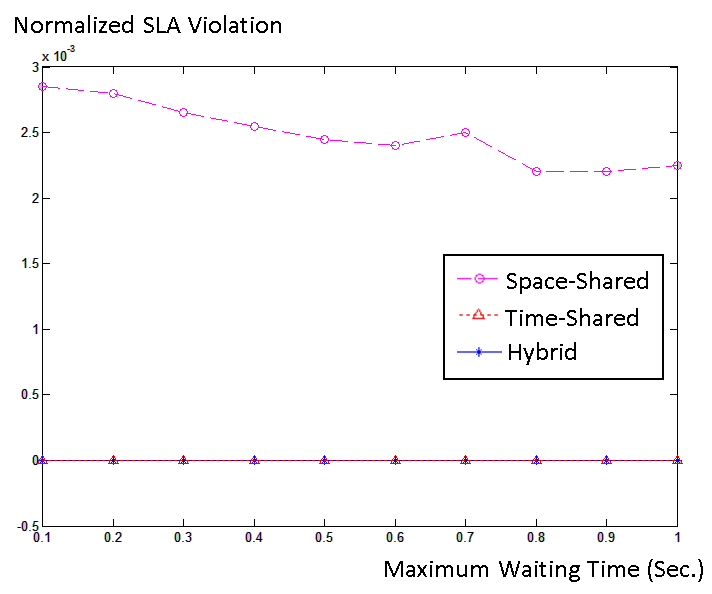

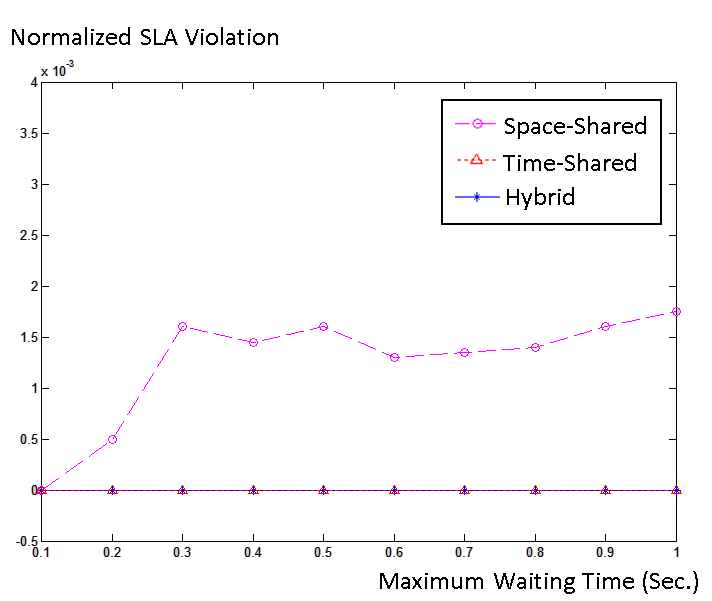

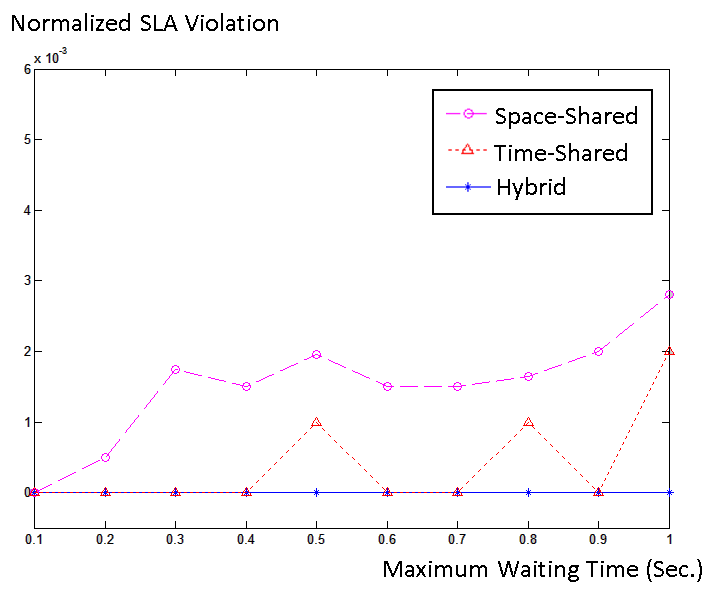


Figure 3a: b=0.99 Figure 3b: b=0.9 Figure 3c: b=0.8


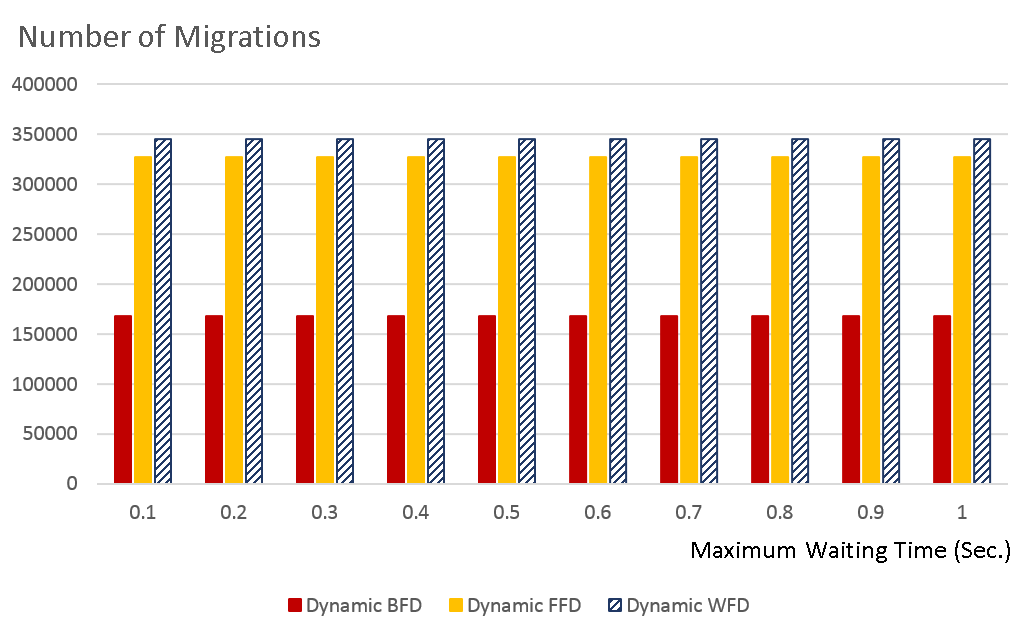


Figure 4


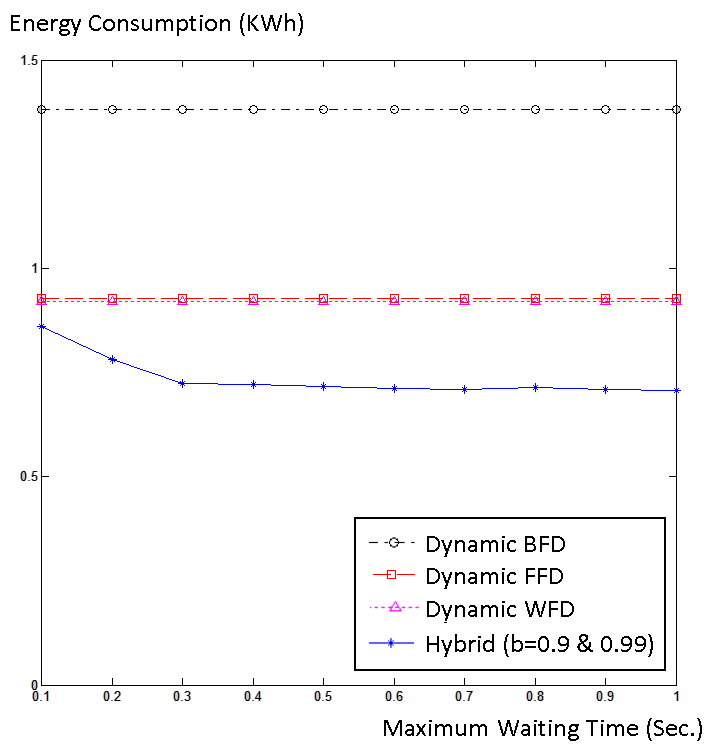

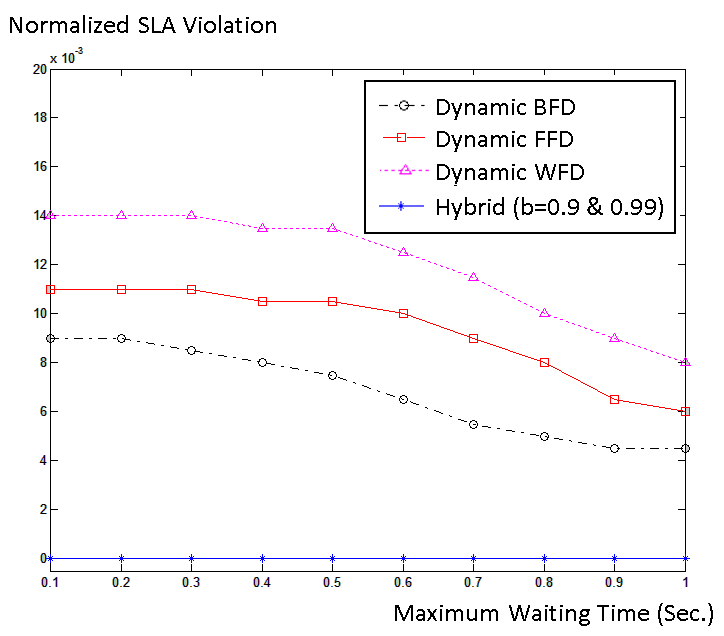


Figure 5 Figure 6
