# Supplementary material for: A static VM placement and hybrid job scheduling model for green data centers
Source: PLoS One. 2020 Aug 13;15(8):e0237238. doi: 10.1371/journal.pone.0237238 (PMC7425884; doi:10.1371/journal.pone.0237238)
Supplement: S1 Data — (ZIP) [file pone.0237238.s001.zip › MyProject/ReadMe.docx]

Dear Reader,

We have implemented our work with and without utilizing Matlab functions. If your computer is not strong enough to execute our heavy project with Matlab you can use the codes that do not require Matlab to run.

Our project requires cloudsim-3.0.3 which you can download from here: <https://github.com/Cloudslab/cloudsim/releases/tag/cloudsim-3.0.3>

Before running our code, have two points in mind:

1. Do not forget to change the value of the “path” variable in the Main file of each project to the location of CloudSim on your computer. (In each Main.java file, the first variable is ‘String path’ which should be equal to the path of cloudsim on your computer.
2. Since we have used many random numbers in our projects, each time you execute a project the result might slightly change. To get an accurate answer you must execute it several times and find the average. For instance, we executed each project about 10 times and calculated their average to achieve high accuracy.

Hope you enjoy our codes,

Best ☺
