# Supplementary material for: A static VM placement and hybrid job scheduling model for green data centers
Source: PLoS One. 2020 Aug 13;15(8):e0237238. doi: 10.1371/journal.pone.0237238 (PMC7425884; doi:10.1371/journal.pone.0237238)
Supplement: S1 Data — (ZIP) [file pone.0237238.s001.zip › MyProject/withMatlab/ReadMe.docx]

To execute the codes:

1. Install Matlab
2. Install a JDK that goes with your version of Matlab.

We worked with Matlab R2013a and JDK 7 on windows 7.

1. Download CloudSim 3.0.3 (<https://github.com/Cloudslab/cloudsim/releases/tag/cloudsim-3.0.3>).
2. Copy our MyProject folder to the examples folder of cloudsim. In other words you must have: cloudsim-3.0.3\examples\MyProject
3. Add these variables to the environmental variables of your OS:

| **variable** | **value** |
| --- | --- |
| path | The path to the bin file of your JDK. For example: C:\Program Files\Java\jdk1.7.0_80\bin; |
| CLASSPATH | The path to the bin file of your JDK. For example: C:\Program Files\Java\jdk1.7.0_80\bin;  <Path to the cloudsim folder>\cloudsim-3.0.3\jars\*;  <Path to the cloudsim folder>\cloudsim-3.0.3\examples; |
| JAVA_HOME | Path to your JDK for example: C:\Program Files\Java\jdk1.7.0_80 |
| MATLAB_JAVA | Path to the jre folder of your JDK, for example:  C:\Program Files\Java\jdk1.7.0_80\jre |

1. Now, if you open Matlab and enter >>version -java you must get the same version of your JDK.
2. Now execute a cloudsim example. Open command prompt and enter these two commands:

javac <path to cloudsim folder>\ cloudsim-3.0.3\examples\org\cloudbus\cloudsim\examples\CloudSimExample1.java

java org.cloudbus.cloudsim.examples.CloudSimExample1

1. Open the classpath.txt of your Matlab. (Something like this: C:\Program Files\MATLAB\R2013a\toolbox\local\classpath.txt) and add these paths to the end of the file:

<path to cloudsim folder>\cloudsim-3.0.3\jars\cloudsim-3.0.3.jar

<path to cloudsim folder>\cloudsim-3.0.3\jars\cloudsim-3.0.3-sources.jar

<path to cloudsim folder>\cloudsim-3.0.3\jars\cloudsim-examples-3.0.3.jar

<path to cloudsim folder>\cloudsim-3.0.3\jars\cloudsim-examples-3.0.3-sources.jar

<path to cloudsim folder>\cloudsim-3.0.3\examples

1. Close Matlab and reopen it to execute a cloudsim example in Matlab. In Matlab write these two commands:

>> o = org.cloudbus.cloudsim.examples.CloudSimExample1

>> javaMethod ('main', o, '')

You should be able to run cloudsim example.

1. Now download “matlabcontrol-3.1.0.jar” form here (Do not download matlabcontrol-4.1.0.jar file, it does not work): <https://github.com/jakaplan/matlabcontrol/releases/tag/v3.1.0>

(it is included in MyProject\withMatlab too).

1. locate matlabcontrol-3.1.0.jar at cloudsim-3.0.3\jars and add the path to the classpath.txt file of Matlab. In other words, add this line to the end of the classpath.txt file of Matlab as well (look at step 8):

<path to cloudsim folder>\cloudsim-3.0.3\jars\matlabcontrol-3.1.0.jar

1. Now, to execute any project in our MyProject\withMatlab folder, first create the class files of the java files by entering for example the code below in command prompt:

>javac <path to cloudsim folder>\cloudsim-3.0.3\examples\MyProject\withMatlab\Hybrid\Main.java

The command above creates the class files of the java files of our Hybrid project. You can do the same thing for SpaceShare, TimeShare, dynamicWd, dynamic Ffd, dynamicBfd too.

1. Next, open Matlab and for example for the Hybrid project enter:

>> o = MyProject.withMatlab.Hybrid

>> javaMethod ('Main', o, '')

1. Congrats!!
