# Supplementary material for: A static VM placement and hybrid job scheduling model for green data centers
Source: PLoS One. 2020 Aug 13;15(8):e0237238. doi: 10.1371/journal.pone.0237238 (PMC7425884; doi:10.1371/journal.pone.0237238)
Supplement: S1 Data — (ZIP) [file pone.0237238.s001.zip › MyProject/withoutMatlab/ReadMe.docx]

To execute the codes:

1. Install JDK.
2. Download CloudSim 3.0.3. (<https://github.com/Cloudslab/cloudsim/releases/tag/cloudsim-3.0.3>)

We worked with JDK 7, windows 7, and cloudsim 3.0.3.

1. Copy our MyProject folder to the examples folder of cloudsim. In other words you must have: cloudsim-3.0.3\examples\MyProject
2. Add these variables to the environmental variables of your OS:

| **variable** | **value** |
| --- | --- |
| path | The path to the bin file of your JDK. For example: C:\Program Files\Java\jdk1.7.0_80\bin; |
| CLASSPATH | The path to the bin file of your JDK. For example: C:\Program Files\Java\jdk1.7.0_80\bin;  <Path to the cloudsim folder>\cloudsim-3.0.3\jars\*;  <Path to the cloudsim folder>\cloudsim-3.0.3\examples; |
| JAVA_HOME | Path to your JDK for example: C:\Program Files\Java\jdk1.7.0_80 |

1. Now you can execute our projects. For example, open command prompt and enter these two commands to execute the Hybrid project:

javac <path to cloudsim folder>\ cloudsim-3.0.3\examples\MyProject\withoutMatlab\Hybrid\Main.java

java MyProject.withoutMatlab.Hybrid.Main

The above commands run the Hybrid project. You can do the same thing for the SpaceShare, TimeShare, dynamicWfd, dynamicFfd, and dynamicBfd as well.

1. Congrats!!
